# methylPipe and compEpiTools: a suite of R packages for the integrative analysis of epigenomics data

**DOI:** 10.1186/s12859-015-0742-6

**Published:** 2015-09-29

**Authors:** Kamal Kishore, Stefano de Pretis, Ryan Lister, Marco J. Morelli, Valerio Bianchi, Bruno Amati, Joseph R. Ecker, Mattia Pelizzola

**Affiliations:** Center for Genomic Science of IIT@SEMM, Istituto Italiano di Tecnologia (IIT), Milano, 20139 Italy; Australian Research Council Centre of Excellence in Plant Energy Biology, The University of Western Australia, Perth, WA 6009 Australia; Genomic Analysis Laboratory, The Salk Institute for Biological Studies, La Jolla, CA 92037 USA; Department of Experimental Oncology, European Institute of Oncology (IEO), Milano, 20139 Italy; Howard Hughes Medical Institute, The Salk Institute for Biological Studies, La Jolla, CA 92037 USA

**Keywords:** Epigenomics, DNA methylation, Histone marks, Integrative biology, High-throughput sequencing

## Abstract

**Background:**

Numerous methods are available to profile several epigenetic marks, providing data with different genome coverage and resolution. Large epigenomic datasets are then generated, and often combined with other high-throughput data, including RNA-seq, ChIP-seq for transcription factors (TFs) binding and DNase-seq experiments. Despite the numerous computational tools covering specific steps in the analysis of large-scale epigenomics data, comprehensive software solutions for their integrative analysis are still missing. Multiple tools must be identified and combined to jointly analyze histone marks, TFs binding and other -omics data together with DNA methylation data, complicating the analysis of these data and their integration with publicly available datasets.

**Results:**

To overcome the burden of integrating various data types with multiple tools, we developed two companion R/Bioconductor packages. The former, methylPipe, is tailored to the analysis of high- or low-resolution DNA methylomes in several species, accommodating (hydroxy-)methyl-cytosines in both CpG and non-CpG sequence context. The analysis of multiple whole-genome bisulfite sequencing experiments is supported, while maintaining the ability of integrating targeted genomic data. The latter, compEpiTools, seamlessly incorporates the results obtained with methylPipe and supports their integration with other epigenomics data. It provides a number of methods to score these data in regions of interest, leading to the identification of enhancers, lncRNAs, and RNAPII stalling/elongation dynamics. Moreover, it allows a fast and comprehensive annotation of the resulting genomic regions, and the association of the corresponding genes with non-redundant GeneOntology terms. Finally, the package includes a flexible method based on heatmaps for the integration of various data types, combining annotation tracks with continuous or categorical data tracks.

**Conclusions:**

methylPipe and compEpiTools provide a comprehensive Bioconductor-compliant solution for the integrative analysis of heterogeneous epigenomics data. These packages are instrumental in providing biologists with minimal R skills a complete toolkit facilitating the analysis of their own data, or in accelerating the analyses performed by more experienced bioinformaticians.

**Electronic supplementary material:**

The online version of this article (doi:10.1186/s12859-015-0742-6) contains supplementary material, which is available to authorized users.

## Background

In recent years, a wealth of studies including large-scale epigenomics data have been published. Unprecedented developments in DNA sequencing technology have allowed individual research groups to profile multiple epigenetic marks in higher eukaryotes, such as DNA methylation and histone post-translational modifications. In addition, profiling of the components of the regulatory machinery, including transcription factors, RNAPII and cofactors, and DNaseI-sensitive regions, often accompanies these epigenomics datasets. As a consequence, a multitude of data types, generated by different experimental methods and characterized by specific biases and pitfalls are often combined in the same study. Numerous computational tools have been developed for the analysis of epigenomics data, typically focusing on specific analysis steps and data types [1, 2]. Projects such as Galaxy and Bioconductor have been instrumental for dealing with the complexity of the analysis of these data [3, 4]. While the former is very intuitive to use, it is dependent on a limited set of embedded tools. On the other hand, Bioconductor currently offers more than 900 packages for the analysis of high-throughput data; however, it requires greater computational experience for the identification and use of the available resources. Therefore, simple and comprehensive tools for an integrative analysis of these various data types are missing, slowing down the construction of pipelines by bioinformaticians, and leaving biologists generating high-throughput sequencing data dependent upon the expertise of other computational scientists.

To fill this gap, we have developed two companion software packages for the integrative analysis of the most common epigenomics data types. These tools offer easy access to features commonly requested by biologists, providing a complete, user-friendly toolkit for the comprehensive analysis of the data they generate. Moreover, these packages facilitate the execution of relevant tasks and the construction of complex pipelines for bioinformaticians. The former, methylPipe, is tailored to the analysis of high- or low-resolution DNA methylomes in multiple species, accommodating (hydroxy-)methyl-cytosines in both CpG and non-CpG sequence context. The analysis of multiple whole-genome bisulfite sequencing experiments is supported, while maintaining the ability of integrating targeted genomic data. The latter, compEpiTools, seamlessly incorporates the results obtained with methylPipe and supports their integration with other epigenomics data. It provides a number of methods to score these data in regions of interest, leading to the identification of enhancers, lncRNAs, and RNAPII stalling/elongation dynamics. Moreover, it allows a fast and comprehensive annotation of the resulting genomic regions, and the association of the corresponding genes with non-redundant GeneOntology terms. Finally, the package includes a flexible method based on heatmaps for the integration of various data types, combining annotation tracks with continuous or categorical data tracks.

Methods and functions available in the methylPipe and compEpiTools packages will be indicated in italic throughout the text and described in the following sections.

## Implementation

The methylPipe and compEpiTools R packages were developed in compliance with the most common Bioconductor infrastructures. Specifically, classes inheriting from the SummarizedExperiments and the GRanges classes are used to represent DNA methylation and other epigenomics data, respectively [5]. The TxDB and BSgenome classes (available for an extensive set of organisms) are adopted as a reference for genome sequences and gene models, respectively. Therefore, methods and functions within these packages can easily be combined with tools available in other Bioconductor packages for up- or down-stream analysis steps.

**Fig. 1:**
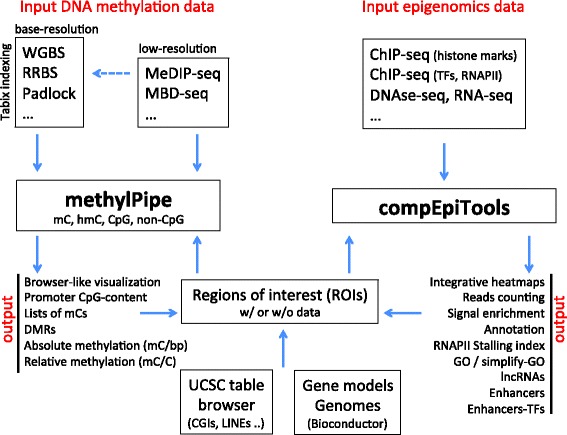
Diagram describing input and output for the methylPipe and compEpiTools R packages. Most typical input data and output are listed for both packages. Regions of interest (*ROIs*) might be both input and output for these tools. For example, input ROIs can be generated in R based on the UCSC table browser or can be based on Bioconductor gene models or reference genome-sequence packages. Output ROIs are generated by methylPipe and compEpiTools and can typically feedback on the same tools as a new set of genomic regions to be investigated, often associated with scores or more complex data. Abbreviations: differentially methylated regions (*DMRs*); methyl-cytosine (*mC*); CpG Islands (*CGIs*); GeneOntology (*GO*); long non-coding RNAs (*lncRNAs*); transcription factors (*TFs*). The dashed arrow identifies a computational step that can be covered with additional tools (see the text for details)

Some of these datasets can be particularly large: for example, data resulting from whole-genome bisulfite (WGBS) experiments in human cells. In order to accommodate studies including multiple WGBS without affecting performance (in terms of speed and required memory), in the packages we developed, the data are maintained on the disk as indexed and compressed flat files [6]. The code is parallelized in order to minimize the computational time for the most demanding tasks, as in the case of the identification of differentially methylated regions. Figure 1 illustrates the overall design along with the main input and output of the methylPipe and compEpiTools packages.

As required in Bioconductor, each individual method and function is accompanied by specific documentation and working examples. Moreover, each package contains a vignette to interactively demonstrate the software key functionalities and a typical workflow. In addition, a supplemental vignette is provided (see online Additional file 1) illustrating a workflow built on publicly available DNA methylation and epigenomics data, where a number of the provided features from both packages are exemplified.

## Results and discussion

### methylPipe features

The base-resolution DNA methylation data used as input can be provided as tabular text files containing, for each profiled cytosine, the genomic positions and the number of reads with C or T (depending whether the cytosine was protected or converted by the action of sodium bisulfite treatment [7], respectively). Alternatively, these data can be generated providing the path to SAM files of aligned reads such as, but not limited to, the alignment files obtained with the popular Bismark aligner [8]. Data are stored as Tabix-indexed compressed files [6] enabling compact representation and fast access to post-alignment processed DNA methylation data (*BSprepare* function). For example, the size of a compressed WGBS experiment for IMR90 and H1 human cell lines is 269 MB and 380 MB, respectively. Not only the limited size of this file results in a reduced disk space requirements (the size of the uncompressed flat files is 372 MB and 554 MB, respectively): these data can also be directly accessed from the disk, thanks to the Tabix indexing, further saving on the memory usage. Through this strategy methylPipe can easily accommodate data from multiple WGBS experiments or any combination of WGBS and targeted base-resolution datasets. In addition to the methylPipe package, the complete set of mCs mapped in the IMR90 and H1 cell lines in the first human base-resolution DNA methylomes profiled by Lister and colleagues [9] are included in the ListerEtAlBSseq Bioconductor metadata package. The WGBS data available in this package were processed, compressed and indexed with Tabix through methylPipe and can directly be accessed using the package functionalities (see below for details).

When post-alignment tabular DNA methylation data are processed in methylPipe through the *BSprepare* function, the confidence of calling a mC is determined for each C through a binomial test [9]. Briefly, sodium bisulfite treatment of DNA specifically converts unmethylated C to U (ultimately read as T) without affecting methylated C. For a given cytosine in the reference genome, the more sequencing reads have a C, the higher is the likelihood of that C being methylated. The binomial test is performed taking into account both the bisulfite conversion rate, which is typically calculated by sequencing of an unmethylated spike-in, and the sequencing error rate. The resulting multiple-testing corrected p-values are stored on the disk in the Tabix compressed and indexed file, and are available in methylPipe through the *BSdata* class. This class has methods to easily access and filter the base-resolution data based on sequencing depth and statistical significance of the mC call. While using the binomial test to measure the confidence of a methylation event is straightforward in case of cell lines or very pure cell populations, its interpretation could be less direct in case contaminants or subpopulations with mixed epigenetic states are present in the sample. In those cases, the number of reads with C (#C, supporting the methylation call at a given cytosine), the number of reads with T (#T, not supporting the methylation call), and the combined methylation level summary #C/(#C + #T) are available in the *BSdata* class and should be used for evaluating this heterogeneity. Further methods are being developed to resolve distinct epigenomes in mixed population [10, 11].

DNA methylation data are typically profiled over a set of regions of interest (ROIs) such as CpG Islands or gene promoters. A class inheriting from the Bioconductor SummarizedExperiment class was defined for this task (*GEcollection* class). This class has data slots specific for the absolute and relative density of DNA methylation events, which can be populated with the *profileDNAmetBin* method. The absolute methylation level of a genomic region (or bins thereof) is determined as the number of mCs per base-pair, while the relative methylation level is determined as the proportion of mCs over the total number of potential methylation sites in that region. While the methylPipe *GEcollection* class is designed to profile DNA methylation in a set of genomic regions for a single sample, the class *GElist* can be conveniently used to collect the same information for a number of samples and pass it on to other methylPipe or compEpiTools methods.

Particular attention was dedicated to the strategy used for incorporating base-resolution information about unmethylated and uncovered (unsequenced) cytosines. While unmethylated Cs are the vast majority of the cytosines in all profiled genomes [12], the amount of uncovered Cs depends on the experimental technique adopted. Different techniques are available for the acquisition of base-resolution DNA methylation profiles. These can target the whole genome (WGBS) or only a subset of it, focussing on CpG-rich (Reduced Representation of Bisulfite Sequencing, RRBS [13]) or custom regions (such as the approach based on padlock probes [14]). In WGBS, most of the Cs are profiled, thus there is a limited number of uncovered Cs, and a majority of unmethylated Cs. On the other hand, RRBS or padlock experiments only cover a limited portion of the genome, resulting in data where few unmethylated Cs are vastly outnumbered by a large majority of uncovered Cs. As a trade-off between these extremes, we decided to include in the BSdata class only the cytosines where at least one sequencing read has a C, supporting the methylation call. In addition, we provide a function to generate a GRanges including all the unmapped regions (with no sequence data) based on a BAM file, as most of the uncovered cytosines tend to occur in a limited number of refractory regions in WGBS experiments or in a relatively small number of regions complementary to the RRBS or padlock targeted regions. In this way, (i) considering Cs with #C >0 and (ii) having the list of the regions containing uncovered Cs, we can exactly recover the methylation status for each C in the genome as methylated (at a significant or not significant level), unmethylated or unmapped. Importantly, this piece of information is critical for the identification of the differentially methylated regions by methylPipe. Eventually, this results in a compression of WGBS experiments in relatively small files, while maintaining the ability of efficiently accommodating and integrating experiments with any level of genome targeting.

The *findDMR* function uses the Wilcoxon or Kruskal-Wallis paired non-parametric tests for the identification of differentially methylated regions (DMRs) by comparing the mC methylation levels between either two groups of samples or multiple samples, respectively Briefly, the algorithm adopts a dynamic sliding window approach that identifies regions suitable for testing depending on mC density and relative distance, and possibly excluding regions with no, or negligible, variation between groups. Specifically:

(i) A cytosine identified as methylated by the binomial test, having a minimum sequencing depth and a minimum level of methylation difference among the considered samples is identified as the seed (all these cutoffs are user-defined).
(ii) Downstream Cs, satisfying the same criteria, within a maximum distance (D) from the seed are then considered. A minimum number of Cs (data points) is required within that window, while the method allows a maximum number of missing data (unmapped Cs).
(iii) Next, the methylation level of the considered Cs is compared between the samples using the statistical tests described above.
(iv) The first mC call downstream of the position of the first seed C incremented of D/2 bp is considered as the seed of the next window and the process is repeated from (i).

Alternatively, the *findDMR* function works with the average methylation levels in discrete genomic regions. This could be useful in case of non-CpG methylation events, which are more unevenly distributed in the genome with respect to mCpGs. Importantly, it is possible to upload GRanges with a list of Cs associated to known SNPs, which could confound the DMR analysis; these Cs are discarded from the analysis. Moreover, when comparing differentiated to pluripotent cells, we suggest to include a GRanges object defining the partially methylated domains (*findPMDs*), defined as large regions of partial methylation that could cover up to 30 % of their genome, typically found in differentiated cells [9]. These regions are, by definition, differentially methylated when compared to pluripotent cells, and should be skipped in the DMR analysis presented here, which is targeted to smaller differential regions. Eventually, the *consolidateDMRs* function applies a multiple-testing correction to p-values, and significant genomic regions closer than a user-adjustable threshold are merged (their corresponding p-values are combined using the Fisher’s method).

The *findDMR* method was proven useful for the identification of DMRs in a number of studies [15, 16], resulting in the identification of genomic regions confirmed by other independent studies [17–20]. In addition, the method was successfully adopted for the simultaneous analysis of hundreds of *A. thaliana* WGBS methylomes [21, 22], which are characterized by mosaic DNA methylation patterns [23]. Thus, methylPipe has already been shown to be effective not only in the analysis of very large datasets, but also in managing DNA methylomes of various species, and in particular DNA methylomes with peculiar mC patterning compared to mammals. We made a comparison among several tools for the identification of DMRs and the results are shown in the Additional file 2.

Recently, an additional type of cytosine methylation was discovered, the 5-hydroxy-methylcytosine (hmC), which was proven to be a critical intermediary in active de-methylation pathways [24]. Bisulfite sequencing experiments do not distinguish hmC from mC. Specific experimental methods for the identification of this mark at the base-resolution were developed, and MLML is a popular computational method for a first analysis of these data [25]. methylPipe includes the *process.hmc* function to parse the MLML output and create a BSdata object specific for the hmCs data, which can then be combined with any other kind of DNA methylation data using the package functionalities.

A wide array of alternative methods providing low-resolution DNA methylation estimates are available, including MeDIP- or MBD-seq; these assays are based on the binding of mCs by a specific antibody or methyl-binding protein, respectively. Computational methods are available to convert these low-resolution data into high-resolution estimates [26–28], which can be imported in methylPipe as if they were native high-resolution bisulfite data. Alternatively, low-resolution data describing the methylation of genomic regions can be incorporated in methylPipe at the level of *GEcollection* and *GElist* objects, which were designed to summarize mC density in genomic regions.

Finally, the *plotMeth* method was developed to visualize DNA methylation base-resolution data, as well as their summary over genomic regions (or low-resolution DNA methylation data) along with gene models and other *-omics* data or annotation tracks. This method takes advantage of the Gviz Bioconductor library and allows a genome-browser like visualization of a specific genomic region.

### compEpiTools features

compEpiTools functionalities can be grouped in three main categories: (i) computing several read counts metrics in genomic regions, (ii) performing functional/genomic annotation, and (iii) integrated visualizing of heterogeneous data-types.

The compEpiTools package facilitates several typical operations related to the quantification of the sequencing signal in a set of genomic regions. The base-level or overall count of reads within a set of regions can be determined starting from BAM files, using the *GRbaseCoverage* and *GRcoverage* methods, respectively. The resulting counts can be normalized by library size and/or region length. In addition, specifically for ChIP-seq experiments, the peak summit position and the overall region enrichment given a matched input sample can be determined, using the *GRcoverageSummit* and *GRenrichment* method, respectively. For RNA Polymerase II (RNAPII) ChIP-seq experiments, the *stallingIndex* and *plotStallingIndex* functions are available to compute and visualize the cumulative distribution of the stalling index (SI), thus estimating the degree of RNA Polymerase stalling [29]. The SI is defined as the ratio of the RNAPII signal in the promoter and genebody region. When comparing different samples, the SI could increase significantly because of either an increase at the level of RNAPII in the promoter or a decrease of in the genebody, or because of differential dynamics of RNAPII in these two regions. For this reason, to better dissect the dynamics of differential SI, the cumulative SI distribution is integrated with the analysis of promoters and genebody RNAPII read densities.

To ascertain the biological significance of a set of ROIs (ChIP-seq peaks, DMRs etc.), it is essential to consider their genomic context. With the *GRannotate* method, compEpiTools allows an effortless, rich and fast annotation of genomic regions based on Bioconductor standard annotation libraries derived from the UCSC genome database. Specifically, for each ROI, the distance from the nearest transcription start site and its location are determined; the region location is also annotated based on its overlap with promoters, intragenic and intergenic genomic regions, and the corresponding transcript and gene id(s) and symbol(s) are reported. The resulting GRanges conveniently embed the annotation for all the isoforms that might occur in correspondence of a given ROI. Notably, the user is provided the flexibility to supply additional sources of annotation, which could results from other *-omics* analyses, such as lists of ROIs taken from the literature, or obtained within R using the ucscTableQuery function from the rtracklayer package to access UCSC Tables (e.g. the list of CpG Islands).

Genomic regions can also be annotated in a number of epigenomic relevant states. The *enhancers* method identifies enhancers based on promoter-distal H3K4me1 (indicative of enhancers that could be either active or poised) or H3K27ac (indicative of active enhancers) ChIP-seq peaks, possibly excluding CpG Islands regions. With the *matchEnhancers* method, enhancers can conveniently be matched to the most proximal genes, and possibly stratified based on the association to transcription factor (TF) binding events to study the TF-dependent activity of enhancer regions and putative target regions.

Particularly relevant for DNA methylation is the concept of promoter CpG-content, which is critical for the epigenetic control on the downstream gene expression: variation in the absolute or relative methylation at the level of intermediate or high-CpG density promoters was proven to be associated to differential expression of the downstream gene, compared to low CpG content promoters [30]. The promoter CpG context can be determined with compEpiTools through a sliding window scoring approach proposed by [31], implemented in the *getPromoterClass* function.

The *findLncRNA* function in compEpiTools identifies long non-coding RNAs (lncRNAs) based on their epigenetic signatures. Briefly, the function considers H3K4me3 peaks located far away from promoters and associated with a lower H3K4me1 read density (this requirement allows to avoid enhancer regions) as seeds for the identification of potential lncRNA promoters. Evidence for RNA transcription in the regions downstream and upstream these H3K4me3 peaks is evaluated by computing the read density of RNA-seq, H3K79me2 and/or RNAPII experiments. Random, size-matched genomic regions, (promoters excluded) are used as a background to determine the random expected density of these marks of transcriptional activity. Regions with a signal for these marks greater than the 95^th^ percentile of the background are then selected as putative regions expressing lncRNAs.

Finally, a convenient wrapper (*topGOres*) is provided to perform GeneOntology (GO) enrichment analysis based on a set of Entrez gene ids (query), based on the topGO Bioconductor package. Often, GO terms that are very close in the considered ontology are redundant, thus complicating the interpretation of the results of a GO analysis. For this purpose, compEpiTools provides the *simplifyGO*terms function for pruning poorly informative and redundant enriched terms. The rationale behind this pruning is that often parent and a child enriched-terms point to very similar GO terms, associated to a very similar set of genes. Iteratively, for each enriched term T, the parent of T is searched within the set of enriched terms, based on the specified ontology. If both a parent and a child terms were identified as enriched and if they match to a set of genes overlapping more than a user-adjustable threshold within the query, the parent term is discarded in favour of the more specific child term.

**Fig. 2:**
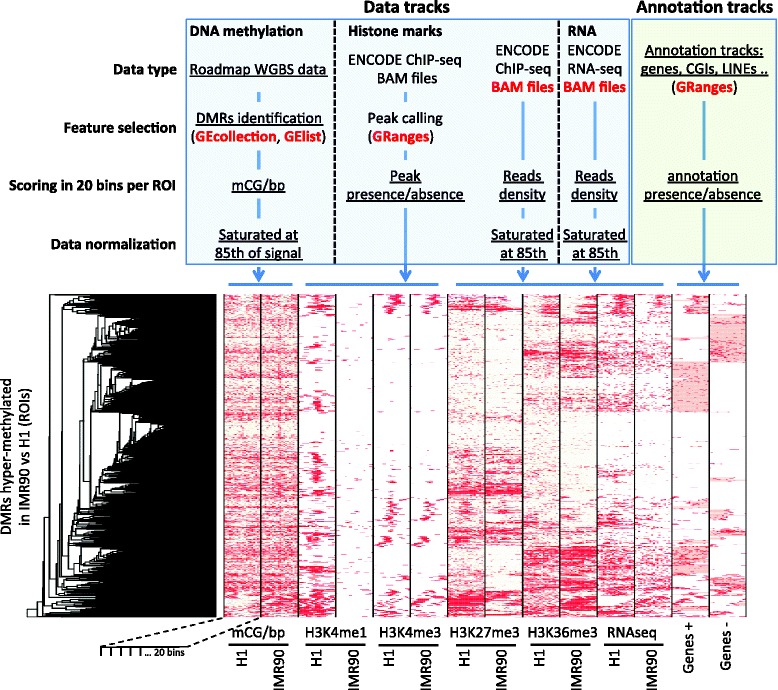
The integrative heatmap generated by the compEpiTools heatmapData and heatmapPlot functions**.** Heatmaps can easily be obtained incorporating any mixture of data and annotation tracks. Heatmap rows represent ROIs, while columns represent tracks profiled over those ROIs (*or bins thereof*). Data and annotation tracks might contain either quantitative (e.g. *normalized reads counts*) or categorical (e.g. *presence/absence of a ChIP-seq peak*) data. If available, the significance of associated data can be incorporated affecting colour brightness. In this example, generated as described in detail in the supplemental material, NIH Roadmap DNA methylation data where visualized together with ENCODE histone marks for a set of differentially methylated regions. ROIs were clustered based on the data available in all the displayed tracks including gene models annotations. The schema on the top of the figure depicts the workflow leading to the heatmap. A set of standard Bioconductor objects, listed in red, is the input for the heatmapData and heatmapPlot compEpiTools functions. The underlined text points to the key analysis steps automatically performed internally to the functions generating the heatmap, calling routines available in the same packages

The integration of heterogeneous data types remains a challenging task, and explorative analyses based on the generation of heatmaps are frequently used to highlight patterns in composite datasets. In our experience, the creation of these heatmaps requires an extensive number of processing steps, especially when applied to datasets composed of heterogeneous data types and annotation tracks, discouraging the repeated use of these tools. Moreover, heatmaps are typically iteratively generated until a satisfactory combination of data tracks, clustering and normalization settings is identified. A powerful and efficient visualization system based on heatmaps is provided in compEpiTools, based on the *heatmapData* and *heatmapPlot* functions. Heatmap rows represent ROIs and columns represent data tracks. Every track can be assigned to any of the supported data types: GRanges, GRanges metadata, BAM files, and GElist and GEcollection objects generated by methylPipe. Thus, any combination of base-resolution or low-resolution DNA methylation data, histone marks, TF binding, RNA-seq expression and genomic annotations, including gene models, is accommodated. Quantile or thresholding-based normalization methods can be independently activated for each track to emphasize patterns in the combined dataset and adjust the signal range of the track (for example to exclude outliers or underweight data tracks that are overall poorly scoring in the ROIs). Clustering of rows can be activated, including data from all or selected tracks. The resolution of the displayed data can be controlled by dividing each ROI in a user-defined number of uniformly-sized bins. Importantly, each track can be supplied with significance scores, which can be used to progressively dim the colour of low-scoring (less significant) hits, while maintaining full brightness for the significant ones. The data matrix underlying the heatmap is returned together with the dendrogram structure, allowing further analysis of the clusters of interest (Fig. 2).

### Computational performance and comparison with other tools

Table 1 provides a comparison of the features offered by methylPipe and compEpiTools with those offered by other computational tools designed for the analysis of epigenomics data. Most of these tools are implemented as R packages, focusing only on the analysis of DNA methylation data. Currently, 38 packages associated with the DNA methylation assay domain are available in Bioconductor. The large majority of these packages were developed for the analysis of data generated with the 450 K Illumina platform, which is able to profile only about 1 % of the cytosines that are typically found in a complete human DNA methylome. Only four of these packages (BiSeq, M3D, bsseq and DSS) could manage WGBS data. These packages provide a very limited subset of the functionalities offered by the methylPipe/compEpiTools packages; most of these tools were developed and tested for the identification of DMRs on RRBS data (Table 1), and claim to be able to analyze WGBS data. We tested whether they could perform three specific tasks we consider necessary in the analysis of WGBS data: (i) uploading a single WGBS dataset and profiling a set of ROIs, (ii) identifying DMRs between 2 conditions, (iii) identifying DMRs between multiple conditions. BiSeq [32] and M3D [33] are designed to upload the entire dataset into memory, and we failed with both in uploading an entire WGBS dataset even when 80 GB of memory was provided (we could only upload and work with data for chromosome 1). Consequently, we were unable to perform any of the three proposed testing operations. Furthermore, bsseq [34] only provides a smoothing-based method to identify DMRs, without offering additional functionalities, and DSS [35] is not specific for DNA methylation data. Neither of these tools delivered satisfactory results: DSS completed the DMR identification in a 2-group comparison in about 3 days, while after the same amount of time bsseq returned an error. Additional packages were included in the comparison (Table 1), while they do not support the analysis of WGBS data (methylKit, MethylSig and Methy-pipe [36–38]), or we encountered the issues descried in Fig 3 legend (WBSA and DMAP [39, 40]). In addition to these Bioconductor packages, few additional stand-alone tools or web-services are available to manage base-resolution DNA methylation data. Among these, only methPipe and radMeth, both developed by the Smith Lab [41, 42], can analyze WGBS datasets and perform DMR analyses. The time needed by these two tools for the identification of DMRs (36 and 90 min, using 1 and 10 cores respectively) is similar or slightly higher than methylPipe (45 min using 10 cores) (Table 1). To our knowledge, the examined tools do not provide an extensive set of supplemental functionalities beyond to those listed in the Table 1. In this regard, methPipe is the only exception, providing additional routines that are complimentary to those offered by methylPipe [41]. In summary, only methPipe and radMeth were able to efficiently complete the proposed tasks with standard resources. Importantly, neither these tools nor the other software packages, limited on the analysis of targeted DNA methylation data, could match the complete set of functionalities offered by methylPipe and compEpiTools (Table 1).

**Table 1:** Comparison of methylPipe and compEpiTools features with functionalities offered by other similar tools

		BiSeq	M3D	Bsseq	DSS	methylKit	methPipe	radMeth	methylSig	WBSA	DMAP	Methy-Pipe
methylPipe	Supporting targeted BS-seq data (e.g. RRBS)	+	+	+	+	+	+	+	+	+	+	+
	Supporting WGBS data	-	-	-	+	-	+	+	-	(a)	(b)	-
	Supporting multi-samples WGBS data	-	-	-	-	-	+	+	-	-	(b)	-
	Non-CpG mCs	-	-	-	-	-	-	-	-	+	-	+
	hmCs	-	-	-	-	+	+	-	-	-	-	-
	Supporting low-resolution DNA methylation data	-	-	-	-	-	-	-	-	-	-	-
	Computing absolute methylation (mC/bp)	-	-	-	-	-	-	-	-	-	-	-
	Computing relative methylation (mC/C)	+	-	+	-	+	+	-	+	+	-	+
	Supporting ROIs binning	-	-	-	-	-	-	-	-	-	-	-
	Pairwise DMR analysis (45’)	+ (NA)	+ (NA)	+ (NA)	+ (3d)	+ (NA)	+ (36’)	+ (90’)	+ (NA)	+ (a)	+	+ (NA)
	Multi-groups DMR analysis	-	-	+	-	-	-	+	-	-	+	-
	Browser-like data plot	-	-	-		-	-	-	+	-	-	-
compEpiTools	Computing Promoter-CpG content	-	-	-	-	-	-	-	-	-	-	-
	Routines for reads counting	-	-	-	-	-	-	-	-	-	-	-
	Determining Signal enrichment	-	-	-	-	-	-	-	-	-	-	-
	ROIs Annotation	+	-	-	-	+	-	-	+	+	+	-
	RNAPII stalling index	-	-	-	-	-	-	-	-	-	-	-
	Non-redundant GO enrichment enrichment	-	-	-	-	-	-	-	-	-	-	-
	Enhancers identification	-	-	-	-	-	-	-	-	-	-	-
	lncRNAs identification	-	-	-	-	-	-	-	-	-	-	-
	Integrative heatmaps	-	-	-	-	-	-	-	-	-	-	-
Ref.		[32]	[33]	[34]	[35]	[36]	[41]	[42]	[37]	[39]	[40]	[38]

The first column lists the key features offered by methylPipe and compEpiTools. Column headers report the tool name and reference. A “+” sign indicates that the feature is provided by a given tool, while a “–“sign indicates that it is not available. The “Pairwise DMR analysis” row includes in parenthesis the time (in minutes or days) needed for a complete WGBS differential analysis between two samples; NA is reported if this analysis is not supported for WGBS data. (a) WBSA is an online web-service imposing a limitation or 2GB for the upload of fastq files, which is clearly insufficient for the analysis of a WGBS dataset; the software can be installed locally although this requires significant effort (requiring Perl, R, MySQL, Java and C compiler) and it is only available for Linux; the analysis of the H1 and IMR90 WGBS was reported by the Authors to be completed in one week. (b) We could not use DMAP (no version details were provided for the code available) at the time of this comparison for the analysis of WGBS data, since an error was returned; a new version was provided to us, which was still requiring details on the restriction enzymes, necessary for the analysis of targeted DNA methylation datasets; eventually it remains unclear to us whether DMAP is able to analyse WGBS data

On the performance side, methylPipe was optimized for the analysis of multiple WGBS datasets. TABIX compression, indexing and computation of binomial tests, which are the steps necessary to create a new *BSdata* object in methylPipe, took 30 min with 1 core (max 4GB RAM peak usage) for the human H1 stem cells [9]. After this data processing step, access to the data is fast: with one core, it can profile 100 human promoters in a sample in about 50 s (max 1GB RAM peak usage). DMRs identification between 2 WGBS samples took 20 min with 1 core on chromosome 1 (max 4GB RAM peak usage), and 45 min with 10 cores for a genome-wide analysis (max 28GB RAM peak usage on a cluster). Finally, the most computationally intense task, i.e. the identification of DMRs among 8 WGBS methylomes [15], took 40 min on chromosome 1 with a single core (max 4.9GB RAM peak usage), proving to be manageable even on a laptop computer. Parallelization is implemented in the package, and it automatically adjusts to the available number of cores and RAM. The same DMR analysis of 8 WGBS methylomes could in fact be completed in a similar time on a cluster by assigning 10 cores.

Most of the functionalities offered by compEpiTools can be run on the order of minutes or less. For example, profiling the normalized number of reads from a GRanges of 40.000 ROIs and a typical BAM ChIP-seq file takes less than 40 s. Dividing each region in a number of bins does not require much additional time, because the binning is performed after the initial count, which is the most time consuming step. Building heatmaps with a dozen of tracks is typically performed in a few minutes, mostly depending on the number of ROIs to be clustered. To achieve maximum efficiency, optimized clustering routines, as implemented in the fastcluster R package, are adopted [43]. The only tool which to our knowledge is comparable in functionality with compEpiTools is the Bioconductor RepiTools package [44]. While RepiTools provides a useful set of tools for the integrative analysis of epigenomics data, mostly focused on statistical testing, integration with gene expression data and visualization, it is tailored to enrichment-based epigenomics data only, and it is unable to provide most of the compEpiTools functionalities listed in Table 1.

## Conclusions

The methylPipe and compEpiTools companion libraries offer a comprehensive system for the integrative analysis of heterogeneous epigenomics data types. methylPipe provides a set of classes, methods and functions that are tailored to DNA methylation high-throughput data, while accommodating data highly different in terms of resolution and genome coverage. To our knowledge, methylPipe is the first software package allowing the analysis and manipulation of multiple WGBS experiments while also being compatible with targeted or low-resolution DNA methylation experiments. Furthermore, compEpiTools includes a series of methods and functions that are commonly used in the integrative analysis of epigenomics, genomics and regulatory datasets. Importantly, compEpiTools is compatible with methylPipe classes, thus allowing an effortless integration of the two packages. Lower-level versions of few of these functionalities are already available albeit dispersed in various Bioconductor packages, such as the routines for counting reads. For these tasks methylPipe and compEpiTools provide simplified and more homogeneous access to lower-level routines, adding an extensive number of new functionalities for DNA methylation and other epigenomics and regulatory data types. Altogether, this suite of packages provides a clear reference entry-point for scientists focusing on the analysis of epigenomics data. This set of tools is currently being successfully used to build pipelines for the most common -*omics* data types. Even more importantly, in our hands this approach is proving to be an excellent resource to effectively provide to experimental scientists with very basic R skills a complete toolkit for the comprehensive analysis of their own generated data.

In conclusion, the Bioconductor-compliant methylPipe and compEpiTools packages provide a comprehensive suite of tools for the integrative analysis of epigenomics data, covering most of the functionalities commonly required in the joint analysis of DNA methylation and epigenomics data.

## Availability and requirements

**Project name:** methylPipe, compEpiTools.

**Project home page:**http://bioconductor.org/packages/methylPipe/.

http://bioconductor.org/packages/compEpiTools/.

http://bioconductor.org/packages/ListerEtAlBSseq/.

**Operating system(s):** Platform independent.

**Programming language:** R (> = 3.1.1).

**License:** GPL (> = 2).

**Any restrictions to use by non-academics:** NA.

## Additional files

Additional file 1:
**methylPipe and compEpiTools supplemental vignette illustrating a workflow built on publicly available DNA methylation and epigenomics data, where a number of the provided features from both packages are exemplified.** (PDF 1156 kb) Additional file 2:
**Comparison of DMRs identified using various tools contains the source code and the results of a comparative analysis of the DMRs identified using various tools.** (PDF 429 kb)

## Abbreviations

mC: Methyl-cytosine
hmC: Hydroxyl-methyl-cytosine
WGBS: Whole-genome bisulfite
ROIs: Regions of interest
RRBS: Reduced representation of bisulfite sequencing
TF: Transcription factor
SI: Stalling index
lncRNAs: Long non-coding RNAs
GO: GeneOntology
DMRs: Differentially methylated regions
