# Supplementary material for: methylPipe and compEpiTools: a suite of R packages for the integrative analysis of epigenomics data
Source: BMC Bioinformatics. 2015 Sep 29;16:313. doi: 10.1186/s12859-015-0742-6 (PMC4587815; doi:10.1186/s12859-015-0742-6)
Supplement: Additional file 1: — methylPipe and compEpiTools supplemental vignette illustrating a workflow built on publicly available DNA methylation and epigenomics data, where a number of the provided features from both packages are exemplified. (PDF 1156 kb) [file 12859_2015_742_MOESM1_ESM.pdf]

# *methylPipe and compEpiTools: a suite of R packages for the integrative analysis of (epi)genomics data*
Supplementary material

August 25, 2015

## Contents


**1 Introduction** **1**

**2 Creation of BSdataSet** **2**

**3 Descriptive statistics of DNA methylation** **2**

**4 Identification of differentially methylated regions** **3**

**5 Genomic location of DMRs** **4**

**6 Distance from TSS and GO enrichment** **5**

**7 Complete Annotation of DMRs** **7**

**8 Integrative visualization of DNA methylation on a region of interest** **8**

**9 Promoters CpG content** **9**

**10 Enhancers** **11**

**11 Generating Heatmap** **13**

**12 Session Information** **18**


## 1 Introduction

**The source code and data used to generate this document is available at** `http://genomics.iit.it/supplementalData/methylPipeSupplementalData`.

This document describes the analysis of ENCODE epigenomics data using the ***methylPipe*** and ***compEpiTools*** companion packages. It illustrates how the combined functionalities offered from the two packages could be used for the integrative analysis of DNA methylation, ChIP-seq, RNA-seq data and genomic annotations.

This document is supplementary material in addition to the Bioconductor vignettes, which are available for both packages, and specific documentation and running examples, which are available for all the functions, methods and classes within those two packages.

## 2 Creation of BSdataSet

First *methylPipe*, *compEpiTools*, *ListerEtAlBSseq* and the genome sequence libraries are loaded. The *ListerEtAlBSseq* package includes complete base-resolution human DNA methylomes (Lister R et al Nature 2009) for two widely studied cell lines: embryonic stem cells (H1) and fetal lung fibroblasts (IMR90). The base-resolution data are stored within *ListerEtAlBSseq* in the form of a *BSdata* object for each cell line. Multiple *BSdata* objects can be conveniently stored in a *BSdataSet* object by specifying group name for each sample either as "C" (control) or "E" (Experiment):

```
library(methylPipe)
library(compEpiTools)
library(BSgenome.Hsapiens.UCSC.hg18)
library(ListerEtAlBSseq)
h1data <- system.file('extdata', 'mc_h1_tabix.txt.gz', package='ListerEtAlBSseq')
h1uncov <- system.file('extdata', 'uncov_GR_h1.Rdata', package='ListerEtAlBSseq')
load(h1uncov)
H1.WGBS <- BSdata(file=h1data, uncov=uncov_GR_h1, org=Hsapiens)
imr90data <- system.file('extdata', 'mc_i90_tabix.txt.gz', package='ListerEtAlBSseq')
imr90uncov <- system.file('extdata', 'uncov_GR_imr90.Rdata', package='ListerEtAlBSseq')
load(imr90uncov)
IMR90.WGBS <- BSdata(file=imr90data, uncov=uncov_GR_imr90, org=Hsapiens)
H1.IMR90.set <- BSdataSet(org=Hsapiens, group=c("C","E"), IMR90_BS=IMR90.WGBS,
                          H1_BS=H1.WGBS)
H1.IMR90.set

## S4 Object of class BSdataSet
##
## BSdata objects contained:

## [1] "IMR90_BS" "H1_BS"

##
## Groups of objects:

## [1] "C" "E"

##
## Associated organism genome:
## Homo sapiens
```

## 3 Descriptive statistics of DNA methylation

*methylPipe* allows quality control of samples by checking summary statistics and assessing sample similarity with correlation and clustering analysis. The `methstats` method takes *BSdataSet* object as an input. Firstly, it summarizes the DNA methylation state of each sample. In addition, it computes pairwise correlation coefficients (Pearson) between the methylation profiles across all the samples in *BSdataSet* object. It outputs scatter plot matrix of correlation coefficients. Finally, it performs (eucledian distance based) hierarchical clustering of samples and outputs the dendrogram. In the example below, the analysis is performed on *BSdataSet* object of artificially replicated H1 and IMR90.

```
stats.set <- BSdataSet(org=Hsapiens, group=c("C","C","E","E"), IMR_1=IMR90.WGBS,
IMR_2=IMR90.WGBS, H1_1=H1.WGBS,H1_2=H1.WGBS)
stats_res <- methstats(stats.set,chrom="chrY",mcClass='mCG', Nproc=1)
stats_res

## $descriptive_stats
##      IMR_1             IMR_2              H1_1              H1_2
##  Min.   :0.0000   Min.   :0.0000   Min.   :0.0000   Min.   :0.0000
##  1st Qu.:0.0000   1st Qu.:0.0000   1st Qu.:0.6360   1st Qu.:0.6360
##  Median :0.0000   Median :0.0000   Median :0.8000   Median :0.8000
##  Mean   :0.1644   Mean   :0.1644   Mean   :0.7463   Mean   :0.7463
##  3rd Qu.:0.2310   3rd Qu.:0.2310   3rd Qu.:0.9580   3rd Qu.:0.9580
##  Max.   :1.0000   Max.   :1.0000   Max.   :1.0000   Max.   :1.0000
##
## $correlation_mat
##            IMR_1      IMR_2       H1_1       H1_2
## IMR_1  1.0000000  1.0000000 -0.1185278 -0.1185278
## IMR_2  1.0000000  1.0000000 -0.1185278 -0.1185278
## H1_1  -0.1185278 -0.1185278  1.0000000  1.0000000
## H1_2  -0.1185278 -0.1185278  1.0000000  1.0000000
```

# 4 Identification of differentially methylated regions

The `findDMR` function is here used to identify differentially methylated regions (DMRs) for cytosines in the CpG sequence context for chromosome 6. IMR90 cells are characterized by extended domains of partial methylation (PMDs), which are absent in pluripotent H1 cells. In order to avoid for these regions to be identified as DMRs, PMDs are provided to the findDMR function, to mask that part of the genome. In this analysis we require for the DMR to contain at least 12 cytosines in a region of maximum length of 1200bp. In addition, the cytosines used as seed for the opening of windows must have a minimum coverage of 5 reads and percent methylation difference larger than 30 between IMR90 and H1.

The `findDMR` function adopts different statistical tests according to the number of groups to be compared. The Wilcoxon-rank test or Kruskal-Wallis non-parametric statistical tests are used in case of pairwise or multi-sample comparisons, respectively. All the evaluated regions are reported in the output with their corresponding statistical significance. The methylation difference is also reported in terms of MethDiffPerc (percentage difference between the mean methylation of experiment and control) and log2Enrichment (log2 of mean methylation of experiment over control) in case of pair wise comparison. Thereafter, the `consolidateDMRs` function is applied to multiple-testing correct the DMRs, consolidate them according to their relative distance, type of DMRs (hypo- or hyper-methylated in the baseline) and thresholds of methylation differences.

In the example below DMRs are identified between H1 and IMR90 having a corrected p-value less than 0.05 and joining regions closer than 100bp. By specifying type="hypo", we focus on the DMRs hypo-methylated in H1 compared to IMR90.

```
PMDs <- read.csv("IMR_PMDs.csv")
PMDs_gr <- GRanges(Rle(PMDs[,1]),IRanges(PMDs[,2],PMDs[,3]))
GRchr6 <- GRanges(Rle('chr6'), ranges=IRanges(start=1, end=length(Hsapiens$chr6)))
chr6_DMRs_12_1200 <- findDMR(object= H1.IMR90.set, ROI=GRchr6, Nproc=1,
                             pmdGRanges=PMDs_gr, MCClass='mCG', coverage=5,
```

```
                              dmrSize=12, dmrBp=1200, eprop=0.3)
hypo.DMRs.conso.chr6 <- consolidateDMRs(DmrGR=chr6_DMRs_12_1200, pvThr=0.05,
                                         GAP=100, type="hypo", correct=TRUE)

## Warning:  closing unused connection 5 (<-localhost:11627)

hypo.DMRs.conso.chr6[1:3]

## GRanges object with 3 ranges and 3 metadata columns:
##       seqnames           ranges strand |    pValue MethDiff_Perc
##          <Rle>        <IRanges>  <Rle> | <numeric>     <numeric>
##   [1]     chr6 [162044, 162602]      * |     0.043       -21.292
##   [2]     chr6 [497174, 497388]      * |     0.044       -18.508
##   [3]     chr6 [502510, 502956]      * |     0.045        -28.85
##       log2Enrichment
##            <numeric>
##   [1]         -0.406
##   [2]         -0.318
##   [3]          -0.55
##   -------
##   seqinfo: 1 sequence from an unspecified genome; no seqlengths
```

# 5 Genomic location of DMRs

It is possible have a quick idea on where the DMRs are in the genome by annotating them. Using the compEpiTools `GRannotateSimple` method one can partition DMRs into those overlapping with promoters, intragenic and intergenic regions.

```
library(TxDb.Hsapiens.UCSC.hg18.knownGene)
txdb <- TxDb.Hsapiens.UCSC.hg18.knownGene
library(org.Hs.eg.db)
DMRs_Simpleanno <- GRannotateSimple(hypo.DMRs.conso.chr6,
                txdb=txdb, upstream=2000, downstream=1000)
```

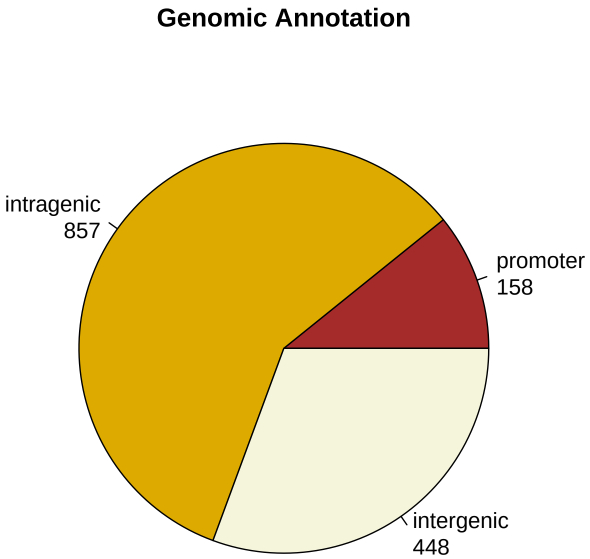


Considering the small size of promoter and gene regions, compared to the intergenic space, the DMRs identified on chr6 are clearly enriched within genes and regulatory regions.

## 6 Distance from TSS and GO enrichment

In this section we illustrate how we could identify genes where promoters are differentially methylated and evaluate their biological functions. The `distanceFromTSS` method can be used to determine Transcription Start Site (TSS) positions for all transcripts in a *TranscriptDb* database and to compute the distance between a set of genomic regions and the most proximal TSS. For example, the first DMR is at a distance of 74498 bp from the TSS of uc003msx.1 transcript of DUSP22 gene (Entrez gene id 56940).

The DMRs proximal to TSS are further selected and the `topGOres` function is applied to the list of associated gene ids to look for over-represented Biological Processes, according to GeneOntology gene annotations. Resulting GeneOntology terms are screened to discard redundant associations using the `simplifyGOterms` function. This is performed taking advantage of the ontology structure and parents-children terms relationships, considering that children terms are by definition more specific than their parents. Specifically, if a given term and its parent(s) are both associated to a similar set of genes (overlap higher than 40 percent for the genes listed in the query in the example below), the parent term(s) can consequently be discarded.

```
DMRs_TSS <- distanceFromTSS(hypo.DMRs.conso.chr6, txdb ,EG2GS=org.Hs.eg.db)
DMRs_TSS[1:3]

## GRanges object with 3 ranges and 7 metadata columns:
##       seqnames           ranges strand |    pValue MethDiff_Perc
##          <Rle>        <IRanges>  <Rle> | <numeric>     <numeric>
##   [1]     chr6 [162044, 162602]      * |     0.043       -21.292
##   [2]     chr6 [497174, 497388]      * |     0.044       -18.508
```

```
##   [3]     chr6 [502510, 502956]      * |     0.045       -28.850
##       log2Enrichment nearest_tx_name distance_fromTSS nearest_gene_id
##            <numeric>     <character>        <integer>     <character>
##   [1]         -0.406      uc003msx.1            74498           56940
##   [2]         -0.318      uc003mtg.1           104575          135458
##   [3]         -0.550      uc003mtg.1            99007          135458
##       nearest_gene_symbol
##               <character>
##   [1]              DUSP22
##   [2]              HUS1B
##   [3]              HUS1B
##   -------
##   seqinfo: 1 sequence from an unspecified genome; no seqlengths

hist(mcols(DMRs_TSS)$distance_fromTSS, breaks= 50, main='DMRs distance from TSS',
      ylab='counts', xlab='bp from the closer TSS')
```

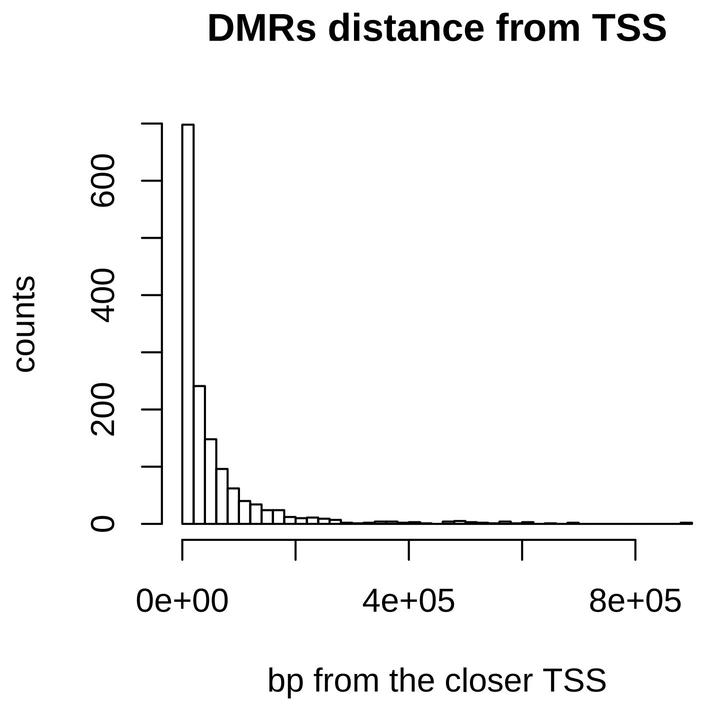


```
inds <- which(mcols(DMRs_TSS)$distance_fromTSS < 2000)
DMRs_nearTSS <- DMRs_TSS[inds]
GOenrich <- topGOres(ids=mcols(DMRs_nearTSS)$nearest_gene_id, ontology='BP',
                     Pthr=1e-03, maxN=5000, minN=5, orgdb=org.Hs.eg.db)

##
## Building most specific GOs ..... ( 10274 GO terms found. )
##
## Build GO DAG topology .......... ( 13787 GO terms and 33379 relations. )
##
```

```
## Annotating nodes ............... ( 16362 genes annotated to the GO terms. )
##
##   -- Classic Algorithm --
##
##   the algorithm is scoring 2562 nontrivial nodes
##   parameters:
##   test statistic:  fisher
```

```
selGOterms= simplifyGOterms(goterms=GOenrich$GO.ID, maxOverlap= 0.4,
                      ontology='BP', go2allEGs= org.Hs.egGO2ALLEGS)
GOenrich= GOenrich[GOenrich$GO.ID %in% selGOterms,]
GOenrich[1:3,]
```

```
##        GO.ID                                          Term Annotated
## 3 GO:0006335 DNA replication-dependent nucleosome assembly        32
## 5 GO:0032776                  DNA methylation on cytosine        36
## 6 GO:0051290                 protein heterotetramerization        36
##   Significant Expected classic                              Genes
## 3           7     0.22 1.8e-09 8354/8356/8360/8362/8366/8367/8368
## 5           7     0.25 4.4e-09 8354/8356/8360/8362/8366/8367/8368
## 6           7     0.25 4.4e-09 8354/8356/8360/8362/8366/8367/8368
```

As expected GeneOntology terms related to cellular development are identified when evaluating genes where TSS-proximal regions are differentially methylated between H1 and IMR90.

## 7 Complete Annotation of DMRs

In addition to genes and promoters, numerous other genomic annotation resources are available for example in the UCSC table browser. These or other user-defined regions of interest, together with genes and promoter annotations can be used with the `GRannotate` method to obtain a rich and comprehensive annotation of a set of genomic ranges. In the following example, CpG Islands (CGIs) annotation are considered in addition to gene structures and promoters, and the CGIs hg18 coordinates are retrieved as GRanges on the fly. In the output each genomic range is mapped to the nearest TSS ('nearest' columns), and placed in the context of the specific location in which it falls ('location' columns) and of the provided CGIs. For example, the second DMR is located in the genebody of both isoforms (uc003mtd.1 and uc003mte.1) of the EXOC2 gene (Entrez Gene ID 55770) and does not overlap a CGI. Overall, 3 percent of the considered hypomethylated DMRs overlap with CGI.

```
library(rtracklayer)
session <- browserSession()
genome(session) <- 'hg18'
query <- ucscTableQuery(session, 'cpgIslandExt')
CGIgr <- as(track(query), 'GRanges')
DMRs_fullanno <- GRannotate(Object=GRmidpoint(hypo.DMRs.conso.chr6)
                            ,txdb=txdb, EG2GS=org.Hs.eg.db,
                            upstream=2000, downstream=1000,
                            userAnn=GRangesList(CGI=CGIgr))
DMRs_fullanno[1:3]
```

```
## GRanges object with 3 ranges and 12 metadata columns:
```

```
##       seqnames           ranges strand |    pValue MethDiff_Perc
##          <Rle>        <IRanges>  <Rle> | <numeric>     <numeric>
##   [1]     chr6 [162323, 162323]      * |     0.043       -21.292
##   [2]     chr6 [497281, 497281]      * |     0.044       -18.508
##   [3]     chr6 [502733, 502733]      * |     0.045       -28.850
##       log2Enrichment nearest_tx_name distance_fromTSS nearest_gene_id
##            <numeric>     <character>        <integer>     <character>
##   [1]         -0.406      uc003msx.1            74777           56940
##   [2]         -0.318      uc003mtg.1           104682          135458
##   [3]         -0.550      uc003mtg.1            99230          135458
##       nearest_gene_symbol          location       location_tx_id
##               <character>       <character>          <character>
##   [1]              DUSP22        intergenic                 <NA>
##   [2]              HUS1B genebody;genebody uc003mtd.1;uc003mte.1
##   [3]              HUS1B genebody;genebody uc003mtd.1;uc003mte.1
##       location_gene_id location_gene_symbol       CGI
##            <character>          <character> <numeric>
##   [1]              <NA>                 <NA>         0
##   [2]      55770;55770          EXOC2;EXOC2         0
##   [3]      55770;55770          EXOC2;EXOC2         0
##   -------
##   seqinfo: 1 sequence from an unspecified genome; no seqlengths

### Percentage of DMRs overlapping with CGIs
ind <- which(mcols(DMRs_fullanno)$CGI==1)
100*length(ind) / length(DMRs_fullanno)

## [1] 3.622693
```

# 8 Integrative visualization of DNA methylation on a region of interest

The `profileDNAmetBin` method is used to profile absolute and relative DNA methylation for a set of regions, generating an object of class *GEcollection*. In this example, DNA methylation is profiled for H1 and IMR90 samples in the identified DMRs. For each genomic region the absolute methylation density (mC/bp), the density of possible methylation sites (C/bp) and the relative methylation level (mC/C) is determined. Both *GEcollection* objects (for H1 and IMR90) are then stored within a *GElist* object. Thereafter, computed densities of methylation event are displayed together with base resolution methylation data and overlaid with annotation information using `plotMeth`. In this example, these tracks are visualized for the OCT4 locus, a key developmental gene, whose promoter is well known to be hypomethylated in pluripotent cells, such as H1.

```
gec.H1.DMRs <- profileDNAmetBin(GenoRanges=hypo.DMRs.conso.chr6,
                    Sample=H1.WGBS, mcCLASS='mCG', nbins=1)
gec.IMR90.DMRs <- profileDNAmetBin(GenoRanges=hypo.DMRs.conso.chr6,
                    Sample=IMR90.WGBS, mcCLASS='mCG', nbins=1)
gel.H1.IMR90.DMRs <- GElist(IMR90.DMRs=gec.IMR90.DMRs,H1.DMRs=gec.H1.DMRs)
H1.IMR90.list <- list(IMR90_BS=IMR90.WGBS, H1_BS=H1.WGBS)
plotMeth(grl=gel.H1.IMR90.DMRs, datatype=c("mC","mC"), yLim=c(.08,.08),
```

```
brmeth=H1.IMR90.list, mcContext="CG",
annodata=GRangesList(DM=hypo.DMRs.conso.chr6),
transcriptDB=txdb, chr="chr6", start=31237000, end=31250000, org=Hsapiens)
```

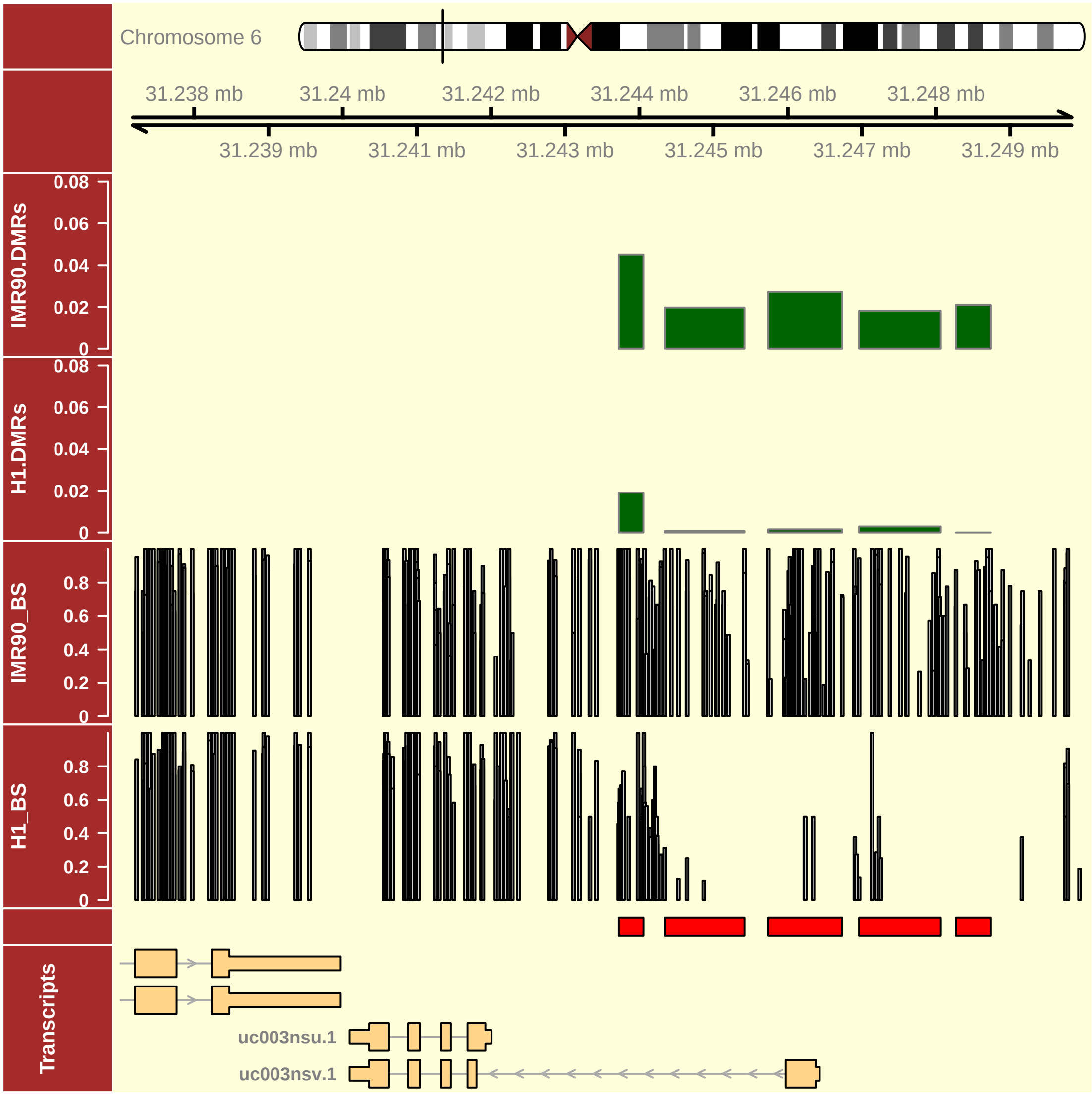


# 9 Promoters CpG content

`getPromoterClass` can be used to classify promoters according to their CpG content. In fact, it was shown that promoters with significantly different CpG content are differently responsive to the presence/absence or even to the level of epigenetic marks such as DNA-methylation. Specifically, promoters with intermediate or high levels or CpG are typically upstream genes differentially expressed when associated with various level of promoter DNA methylation. Rather, the expression of genes under control of promoters with low density of CpG is remarkably insensitive to varying levels of promoter DNA methylation (Koga et al, Genome Research 2009). In the picture below, the promoter CpG content is determined and promoters are classified into low-, intermediate- and high-CpG content. Then, relative DNA methylation (mC/C) is profiled for promoters in the three classes. As can be expected, highCG content promoters have lower methylation level. Whereas, lowCG and intCG promoters have considerably higher relative methylation around the TSS.

```
#### determining the promoter CpG content class
isActiveSeq(txdb) <- c(rep(FALSE,5), TRUE, rep(FALSE, 43))
promoter_chr6 <- getPromoterClass(txdb, Nproc=1, org=Hsapiens, upstream=3000, downstream=3000)
txdb <- restoreSeqlevels(txdb)
highCGprom <- promoter_chr6[mcols(promoter_chr6)$promoterClass=="highCG"]
intCGprom <- promoter_chr6[mcols(promoter_chr6)$promoterClass=="intCG"]
lowCGprom <- promoter_chr6[mcols(promoter_chr6)$promoterClass=="lowCG"]

#### profiling mCpGs in 40 bins for each promoter
chr6_prom_high <- profileDNAmetBin(GenoRanges= highCGprom,
                    Sample=H1.WGBS, mcCLASS='mCG', nbins=40)
chr6_prom_int <- profileDNAmetBin(GenoRanges= intCGprom,
                    Sample=H1.WGBS, mcCLASS='mCG', nbins=40)
chr6_prom_low <- profileDNAmetBin(GenoRanges= lowCGprom,
                    Sample=H1.WGBS, mcCLASS='mCG', nbins=40)

#### extracting the relative methylation level (mC/C)
#### and determining their average profile
meth_high <- binrC(chr6_prom_high)
meth_int <- binrC(chr6_prom_int)
meth_low <- binrC(chr6_prom_low)
meth_high_mean <- apply(meth_high, 2, mean, na.rm=TRUE)
meth_int_mean <- apply(meth_int, 2, mean, na.rm=TRUE)
meth_low_mean <- apply(meth_low, 2, mean, na.rm=TRUE)

#### plotting
plot(meth_high_mean, type='l', lwd=2.5, axes=F, xlab=NA,
     ylab=NA, ylim=c(0,100), col="darkgreen",
     main = "H1 promoters relative methylation level")
points(meth_int_mean, type='l', lwd=2.5, col="orange")
points(meth_low_mean, type='l', lwd=2.5, col="black")
abline(v=20,lty=2)
box()
axis(side = 2)
axis(side = 1, at=c(0,20,40),labels=c("-3 kb","TSS","+3 kb"))
mtext(side = 2, "Relative methylation (mC/C)", line = 3)
legend(5,30, lty=c(1,1), lwd=c(2.5,2.5, 2.5), title="Promoter class",
       legend=c("high CG","int CG","low CG"), col=c("darkgreen","orange","black"))
```

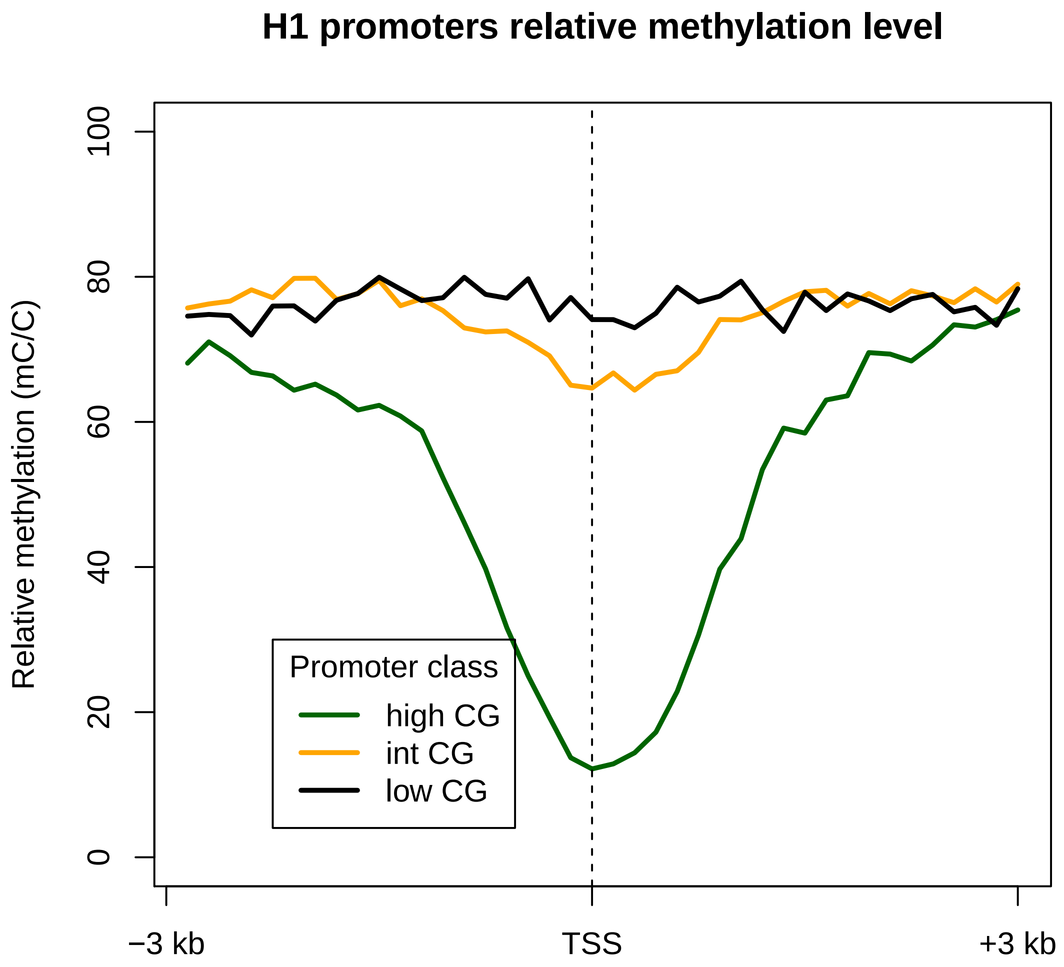


## 10 Enhancers

A number of functions and methods is available to define epigenetically relevant genomic states. The `enhancers` method allows identifying to putative enhancers based on H3K4me1 (thus pointing to enhancers which could be either active or poised) or H3K27ac (thus pointing to active enhancers) marks. In this example, H3K4me1 peaks are processed to identify peaks laying outside gene promoters and not overlapping with CpG Islands to avoid peaks matching to potentially unannotated promoter regions. For an in silico validation of the bona fide identification of these putative enhancers regions we want to verify that, as expected, the H3K4me1/me3 at enhancers is much higher compared to promoter regions. To this purpose, the `GRangesInPromoters` method is used to identify H3K4me1 peaks lying in promoter regions. Having defined enhancer- and promoter-H3K4me1 peaks, the `GRcoverage` method is used to compute the normalized coverage of H3K4me1 and H3K4me3 at these two sets of genomic regions. Thereafter, log2 ratio of the normalized reads density between these two histone marks is computed and its frequency is plotted.

```r
load("H1.4me1_gr.Rdata")
#### location of H3K4me1-based enhancer peaks and H3K4me1 promoter peaks
K4me1_enhancerPeak <- enhancers(gr=H1.4me1_gr, txdb=txdb,
                              upstream= 2000, downstream= 1000, CGIgr=CGIgr)
K4me1_promPeak <- GRangesInPromoters(Object=H1.4me1_gr, txdb=txdb,
                        upstream=2000, downstream=1000, invert=FALSE)

#### density of H3K4me1 and H3K4me3 reads at H3K4me1 enhancer-peaks (EP)
EP_me1 <- GRcoverage(Object=K4me1_enhancerPeak,
              bam="H1.4me1_chr6.bam",
              Nnorm=TRUE, Snorm=FALSE)
EP_me3 <- GRcoverage(Object=K4me1_enhancerPeak,
              bam="H1.4me3_chr6.bam",
              Nnorm=TRUE, Snorm=FALSE)
EP_me1_me3 <- log2(EP_me1/EP_me3)

#### density of H3K4me1 and H3K4me3 reads at H3K4me1 promoter-peaks (PP)
PP_me1 <- GRcoverage(Object=K4me1_promPeak,
              bam="H1.4me1_chr6.bam",
              Nnorm=TRUE, Snorm=FALSE)
PP_me3 <- GRcoverage(Object=K4me1_promPeak,
              bam="H1.4me3_chr6.bam",
              Nnorm=TRUE, Snorm=FALSE)
PP_me1_me3 <- log2(PP_me1/PP_me3)

#### plotting
plot(density(EP_me1_me3, na.rm=TRUE), type="l", xlab="log2(H3k4me1/me3)",
     lwd=2, ylab='Frequency', ylim=c(0,.4), xlim=c(-8,10), col="darkgreen",
     main = "Ratio (H3K3me1/me3) at promoter and enhancer H3K4me1 peaks")
points(density(PP_me1_me3,na.rm=TRUE),type="l", lwd=2, col="red")
legend(5,.4, lty=c(1,1), lwd=c(2,2), title="H3K4me1 peaks",
       legend=c("Enhancer","Promoter"),
       col=c("darkgreen","red"))
```

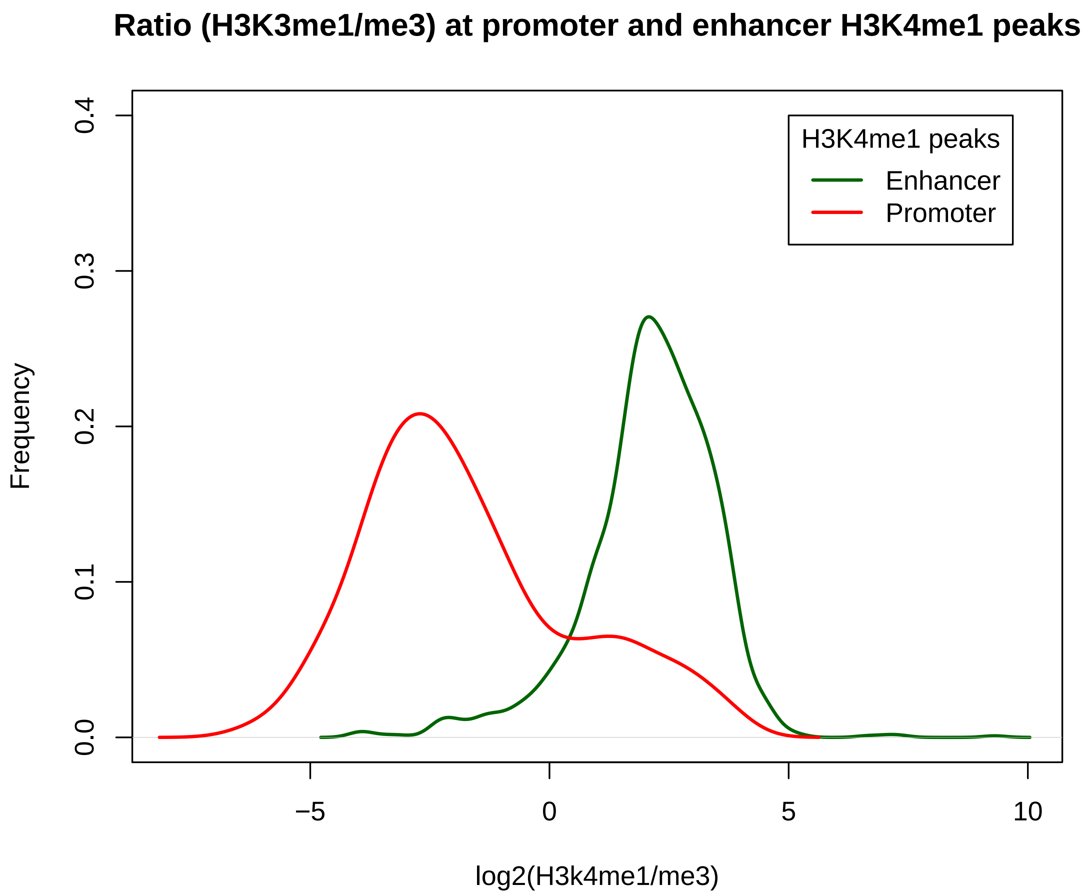


As expected, at enhancer regions, defined as H3K4me1 peaks distal from promoters and not overlapping with CGI, the ratio of H3K4me1 vs H3K4me3 reads is much higher than at H3K4me1 promoter peaks, in which H3K4me3 predominates.

## 11 Generating Heatmap

When integrating heterogeneous data types, heatmaps are often used for explorative analyses and highlight patterns in the combined datasets. compEpiTools provides a powerful and efficient visualization system based on heatmaps. Heatmap rows represent regions of interest (ROIs) and columns represent data tracks. Every track can be freely assigned to any of the supported data types: GRanges, GRanges metadata, BAM files, and various objects generated by methylPipe, thus accommodating any combination of base-resolution or low-resolution DNA methylation data, histone marks, TF binding, RNA-seq expression and genomic annotations, including gene models. A number of normalization methods can be activated independently for each track, to emphasize patterns in the combined dataset and adapt to the track signal range (for example to underweight data tracks that are overall poorly scoring in the ROIs). Clustering of rows can be activated for all or for a selection of the available tracks. The resolution of the data displayed can be defined based on the number of bins that each ROI can be uniformly divided into. Importantly, each track can be provided with significance scores, which can be conveniently used to progressively dim the color of low-scoring (less significant) hits, while maintaining full brilliance for the significant ones. Finally, the data matrix underlying the heatmap is returned together with the dendrogram structure, allowing further analysis and identification of clusters of interest.

There are two main steps for the generation of heatmaps using compEpiTools: (1) the `heatmapData` function has to be called to define the data structure underlying the heatmap. Since multiple bins are often required for each data track, the data are stored into a list, rather than a matrix, and each element of this list is a data track in the final heatmap; a number of normalizing options and the support to various data types allows customizing the counting operation performed by `heatmapData` for each of the final data tracks; (2) the resulting list is passed to the `heatmapPlot` function to display the heatmap, while controlling the coloring scheme, the coloring saturation and the clustering of the rows.

In the following example, the density of DNA methylation is determined 10 kb upstream and downstream of each of the DMRs identified above as hypo-methylated in H1 compared to IMR90 on chromosome 6 (methylation difference greater than 25 percent). For these regions (ROIs), the `heatmapData` function is used to integrate data from various data types. The argument "type" determines how each data type is processed. For incorporating DNA methylation data type can be set to **mC** or **rC** to access absolute or relative methylation, respectively. For H3K4me1 and H3K4me3 data tracks (in both H1 and IMR90 samples) the provided GRanges contain pre-determined ChIP-seq peaks (while they could contain any kind of genomic regions). For these tracks type was set to **gr** so that only GRanges genomic coordinates are considered and represented in the heatmap, as presence or absence of a ChIP-seq peak for each ROI (or bins thereof). For H3K27me3 and H3k36me3 data tracks the aligned reads stored in BAM files (limited to chr6) are provided. The path to the corresponding BAM files are passed to the `heatmapData` function. Consequently, for these tracks type is set to **cov**, so that the reads density (coverage) for these BAM files in the ROIs can be determined. The reads density is further normalized by library size (setting Nnorm argument to TRUE). Finally, regarding the RNA-seq data tracks, the mcols component of the H1 and IMR90 GRanges was used to store pre-computed reads density for these ROIs. This is to show that tracks with any pre-determined quantitative data can be easily added, provided that they match the number of reference ROIs and bins thereof. Type is set to **mcols** to have the data contained in the GRanges mcols component to be directly incorporated in the heatmap. The resolution of the data to be displayed for each track is defined based on the number of bins (nbins) that each ROI is uniformly divided into (20 in this example).

The list resulting from the `heatmapData` function is passed to `heatmapPlot` to display the heatmap. This is convenient in case one would repeat this step testing several plotting and normalization settings, saving the time needed to determine the raw data underlying the heatmap. When calling `heatmapPlot` the data can be normalized independently for each track based on a specific signal percentile (which is set as the maximum saturation value and displayed as 1, corresponding to full red in this example), or based on a specific arbitrarily chosen threshold. In this case a hybrid approach is used. Several tracks for the same data type (for example histone reads density) are temporarily combined and their overall 85th percentile is set as the maximum value. This value is first computed and then assigned to each track using the tnorm `heatmapPlot` argument. This would not have been possible otherwise, since the 85th was computed aggregating various tracks together. The gene annotation information for the forward and reverse strand can be automatically extracted from a *TrascriptDb* object and overlaid in the heatmap, reporting exons in red and intron in pink. This offer the possibility of adding a commonly desired annotation track, using a custom graphic representation to highlight introns and exons. At the same time, this does not prevent to add any other kind of annotation tracks, which can be added without any restriction. The clustering of rows can be activated specifying the index of the tracks to be used for clustering, in this case all of them are used including gene annotation tracks. This option could be useful to direct the clustering to use only a subset of the data or annotation tracks, increasing the flexibility and allowing to emphasize various patterns in the data.

```
### setting the regions of interest (ROIs) as the DMRs hypomethylated in
### H1 compared to IMR90 on chromosome 6 having a the methylation level
### decreased by al least 25% in H1.
```

```r
ind <- which(abs(hypo.DMRs.conso.chr6$MethDiff_Perc) > 25)
DMRs_25 <- hypo.DMRs.conso.chr6[ind]

### Absolute DNA methylation for mC in the CpG context (mCpG/bp)
### is profiled in 10kb regions around the DMRs on chr 6
### dividing each ROI in 20 bins.
start(DMRs_25) <-  start(DMRs_25)-1e4
end(DMRs_25) <-  end(DMRs_25)+1e4
gec.H1_heatmap <- profileDNAmetBin(GenoRanges=DMRs_25,
 Sample=H1.WGBS, mcCLASS='mCG', nbins=20)
gec.IMR90_heatmap <- profileDNAmetBin(GenoRanges=DMRs_25,
 Sample=IMR90.WGBS, mcCLASS='mCG', nbins=20)

#### H1 and IMR90 ENCODE H3K4me1 and H3K4me3 ChIP-seq peaks
#### pre-determined with MACS were saved as GRanges
#### and are loaded into R

load("H1.4me1_gr.Rdata")
load("H1.4me3_gr.Rdata")
load("IMR90.4me1_gr.Rdata")
load("IMR90.4me3_gr.Rdata")

#### The paths to the H1 and IMR90 ENCODE H3K4me1 and H3K4me3 BAM files
#### generated through the BWA aligner are set

H1.27me3_BAM <- "H1.27me3_chr6.bam"
H1.36me3_BAM <- "H1.36me3_chr6.bam"
IMR90.27me3_bam <- "IMR90.27me3_chr6.bam"
IMR90.36me3_bam <- "IMR90.36me3_chr6.bam"

#### pre-computed coverage of H1 and IMR90 ENCODE RNA-seq into thes ROIs
#### was saved into the mcols components of GRanges objects,
#### and the corresponding GRanges loaded into R

load("mrna_h1_gr.Rdata")
load("mrna_imr90_gr.Rdata")

#### a list with an element for each heatmap track is generated

Grl <- list(H1_meth=gec.H1_heatmap, IMR90_meth=gec.IMR90_heatmap, H1_4me1=H1.4me1_gr,
        IMR90_K4me1=IMR90.4me1_gr, H1_K4me3=H1.4me3_gr, IMR90_K4me3=IMR90.4me3_gr,
        H1_K27me3=H1.27me3_BAM, IMR90_K27me3=IMR90.27me3_bam, H1_K36me3=H1.36me3_BAM,
        IMR90_K36me3=IMR90.36me3_bam, RNAseq_H1=mrna_h1_gr, RNAseq_IMR90=mrna_imr90_gr)

#### Type is coherently set to:
#### "mC" for the DNA-methylation tracks
#### "gr" for the ChIP-seq peaks absence/presence
#### "cov" for the ChIP-seq reads densities
#### "mcols" for the pre-computed RNA-seq reads densities
```

```
Type <- c("mC","mC","gr","gr","gr","gr",
    "cov","cov","cov","cov","mcols","mcols")

#### heatmapData is called setting the selected DMRs as ROI (refgr)
#### The hg18 TxDB object is passed to automatically generated
#### gene annotation tracks

combMat <- heatmapData(grl=Grl, refgr=rowRanges(Grl[[1]]), type=Type,
              Nnorm=TRUE, Snorm=FALSE, txdb=txdb, nbins=20)

## [1] "H1_meth"
## [1] "IMR90_meth"
## [1] "H1_4me1"
## [1] "IMR90_K4me1"
## [1] "H1_K4me3"
## [1] "IMR90_K4me3"
## [1] "H1_K27me3"
## [1] "IMR90_K27me3"
## [1] "H1_K36me3"
## [1] "IMR90_K36me3"
## [1] "RNAseq_H1"
## [1] "RNAseq_IMR90"

combMat <- combMat[[1]]

#### 85th percentile of data tracks
#### grouped by data type.
#### This is used to enforce the same threshold
#### for all the tracks of a given data type
#### (DNA methylation, histone marks, RNA-seq)

meth <- unlist(combMat[1:2])
histones <- unlist(combMat[7:10])
gene_exp <- unlist(combMat[11:12])

meth_v <- quantile(meth,.85,na.rm=TRUE)
histones_v <- quantile(histones,.85,na.rm=TRUE)
gene_exp_v <- quantile(gene_exp,.85,na.rm=TRUE)
Tnorm= c(rep(meth_v,2), rep(1,4), rep(histones_v,4),
        rep(gene_exp_v,2), rep(1,2))

#### heatmapPlot is called on the object resulting from
#### the heatmapData function, passing the vector of
#### normalization thresholds (Tnorm) and
#### setting all the tracks (12 plus 2 annotation tracks)
#### to be used for clustering of the ROIs

heatmapPlot(matList=combMat, tnorm=Tnorm, rowLab=FALSE, colLab=TRUE,
        margins=c(8,1), clusterInds=c(1:14))
```

```
## Warning in heatmapPlot(matList = combMat, tnorm = Tnorm, rowLab = FALSE, :  'genes
+' and 'genes -' might be normalized ...
```

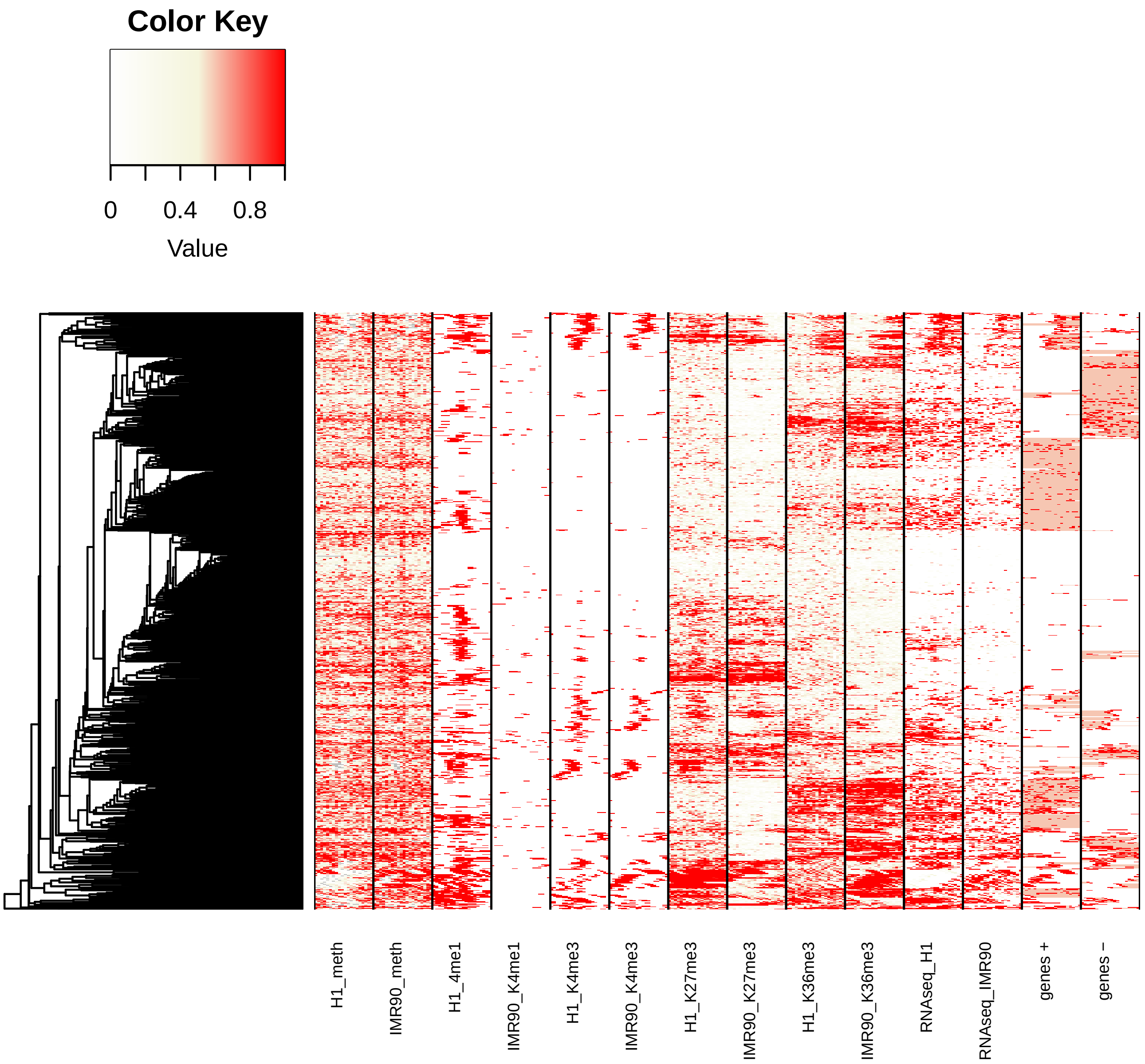

# 12 Session Information

```
sessionInfo()

## R version 3.2.0 (2015-04-16)
## Platform: x86_64-unknown-linux-gnu (64-bit)
## Running under: Debian GNU/Linux 7 (wheezy)
##
## locale:
##  [1] LC_CTYPE=C                 LC_NUMERIC=C
##  [3] LC_TIME=C.UTF-8            LC_COLLATE=C.UTF-8
##  [5] LC_MONETARY=C.UTF-8        LC_MESSAGES=C.UTF-8
##  [7] LC_PAPER=C.UTF-8           LC_NAME=C
##  [9] LC_ADDRESS=C               LC_TELEPHONE=C
## [11] LC_MEASUREMENT=C.UTF-8 LC_IDENTIFICATION=C
##
## attached base packages:
## [1] stats4    parallel  stats     graphics  grDevices utils
## [7] datasets  methods   base
##
## other attached packages:
##  [1] org.Hs.eg.db_3.1.2
##  [2] TxDb.Hsapiens.UCSC.hg18.knownGene_3.1.2
##  [3] GenomicFeatures_1.20.1
##  [4] ListerEtAlBSseq_1.0.0
##  [5] BSgenome.Hsapiens.UCSC.hg18_1.3.1000
##  [6] BSgenome_1.36.3
##  [7] rtracklayer_1.28.7
##  [8] compEpiTools_1.2.5
##  [9] topGO_2.20.0
## [10] SparseM_1.6
## [11] GO.db_3.1.2
## [12] RSQLite_1.0.0
## [13] DBI_0.3.1
## [14] AnnotationDbi_1.30.1
## [15] Biobase_2.28.0
## [16] graph_1.46.0
## [17] methylPipe_1.2.4
## [18] Rsamtools_1.20.4
## [19] Biostrings_2.36.3
## [20] XVector_0.8.0
## [21] GenomicRanges_1.20.5
## [22] GenomeInfoDb_1.4.1
## [23] IRanges_2.2.7
## [24] S4Vectors_0.6.3
## [25] BiocGenerics_0.14.0
## [26] knitr_1.11
##
## loaded via a namespace (and not attached):
```

```
##  [1] splines_3.2.0             gtools_3.5.0
##  [3] Formula_1.2-1             highr_0.5
##  [5] latticeExtra_0.6-26       lattice_0.20-33
##  [7] biovizBase_1.16.0         limma_3.24.15
##  [9] chron_2.3-47              digest_0.6.8
## [11] RColorBrewer_1.1-2        colorspace_1.2-6
## [13] plyr_1.8.3                XML_3.98-1.3
## [15] biomaRt_2.24.0            zlibbioc_1.14.0
## [17] scales_0.2.5              gdata_2.17.0
## [19] BiocParallel_1.2.20       ggplot2_1.0.1
## [21] nnet_7.3-10               Gviz_1.12.1
## [23] proto_0.3-10              survival_2.38-3
## [25] magrittr_1.5              evaluate_0.7.2
## [27] MASS_7.3-43               gplots_2.17.0
## [29] foreign_0.8-65            tools_3.2.0
## [31] data.table_1.9.4          formatR_1.2
## [33] matrixStats_0.14.2        stringr_1.0.0
## [35] munsell_0.4.2             cluster_2.0.3
## [37] lambda.r_1.1.7            caTools_1.17.1
## [39] futile.logger_1.4.1       grid_3.2.0
## [41] RCurl_1.95-4.7            dichromat_2.0-0
## [43] VariantAnnotation_1.14.10 marray_1.46.0
## [45] bitops_1.0-6              gtable_0.1.2
## [47] codetools_0.2-14          reshape2_1.4.1
## [49] GenomicAlignments_1.4.1   gridExtra_2.0.0
## [51] Hmisc_3.16-0              futile.options_1.0.0
## [53] KernSmooth_2.23-15        stringi_0.5-5
## [55] Rcpp_0.12.0               rpart_4.1-10
## [57] acepack_1.3-3.3
```
