# Supplementary material for: methylPipe and compEpiTools: a suite of R packages for the integrative analysis of epigenomics data
Source: BMC Bioinformatics. 2015 Sep 29;16:313. doi: 10.1186/s12859-015-0742-6 (PMC4587815; doi:10.1186/s12859-015-0742-6)
Supplement: Additional file 2: — Comparison of DMRs identified using various tools contains the source code and the results of a comparative analysis of the DMRs identified using various tools. (PDF 429 kb) [file 12859_2015_742_MOESM2_ESM.pdf]

Comparison of the differentially methylated regions (DMRs) or Cytosines (DMCs) identified using various publicly available tools

Regarding the assessment of the DMRs identified by methylPipe, we compared them to those identified by other tools in terms of number of regions, overlap, and annotation. Two important issues complicating this comparison should be considered: (i) some tools only allow identifying differentially methylated sites (DMCs), and (ii) some tools would not work with genome-wide WGBS for the DMR identification. We compared all the tools able to perform DMR (DMC) identification on genome-wide WGBS together with those only able to work with a very limited subset of the data. To this purpose we only considered H1 vs IMR90 methylation data for chromosome 1. The heatmap displayed in **Figure 1** report the number and % overlap between the DMRs (DMCs) identified from the various considered methods (using the *overlapOfGRanges* compEpiTools method). Following each method name is the number of identified regions or sites (e.g. 38924 for methylPipe)

While methylPipe, methylKit and methylSig report a relatively similar number of DMRs, the other methods (radmeth, methPipe and DSS) provide very discordant results: radmeth identified 1.6M DMCs, while DSS only identified 261 individual differential cytosines. methPipe on the other hand identified a high number of individual cytosines (~600k). The overlap is calculated in respect to the method reported on the vertical axis (for example the first heatmap row is about the percentage of methylPipe detected regions confirmed by the other methods). While all this does not reassures about the bona-fide DMR identification it can be noticed that the first three methods have a good agreement. For example 57% and 79% of the methylPipe DMRs are confirmed by methylKit and methylSig, respectively, while methylKit basically provides a subset of the regions identified by methylSig. The comparison with the other tools is greatly complicated by the very discordant number of regions or sites identified.

The DMRs identified by the different methods were also compared in terms of genome annotation (using the *GRannotateSimple* compEpiTools method, **Figure 2**). At least for the methods returning DMRs the results are remarkably similar, in terms of proportion of DMRs falling into different genomic regions (it should be noticed that the whole chr1 data are used here, so no bias would be expected for this analysis, differently from 450k or RRBS data which are enriched in specific genomic regions).

Please see at the end of this document for the specific source code used for these analyses.

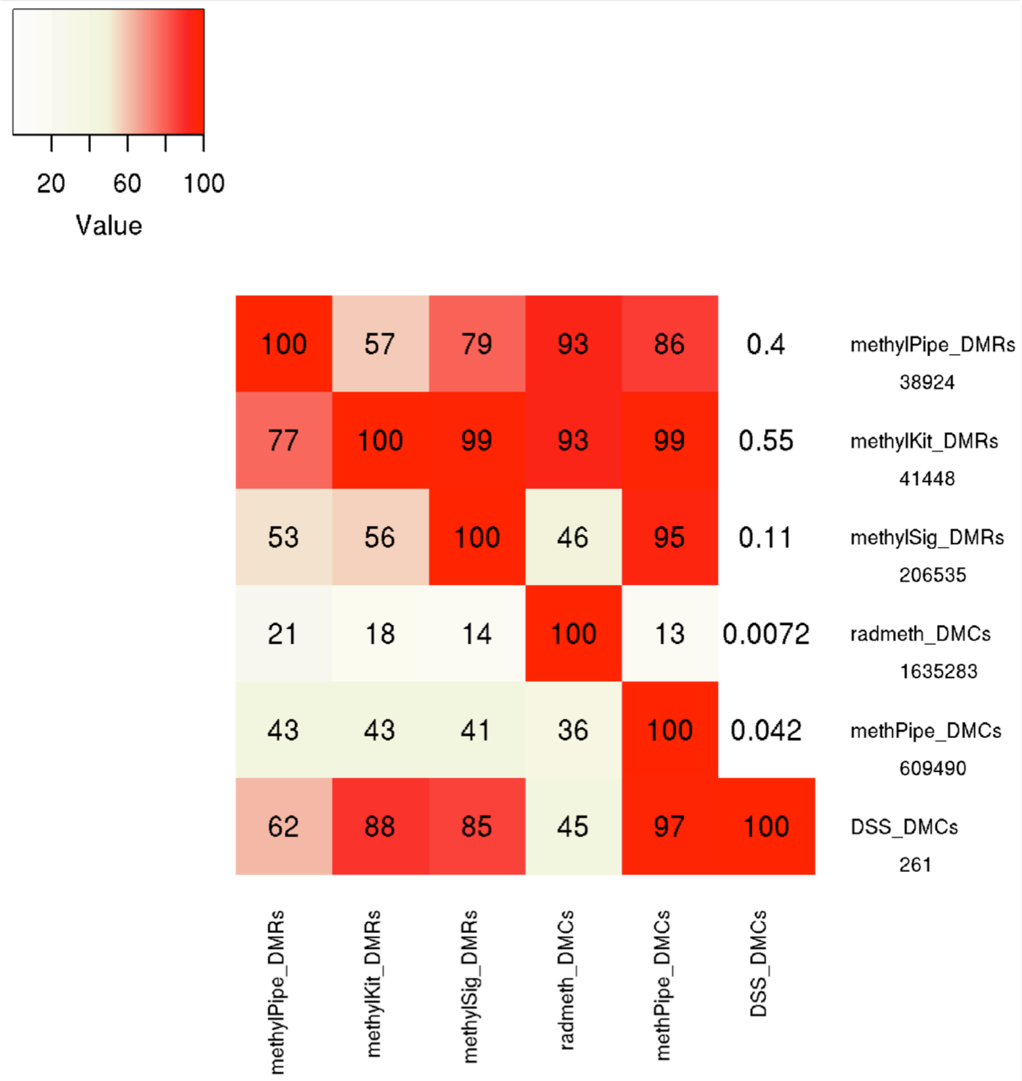

Figure 1 Overlap of differentially methylated regions (DMRs) and differentially methylated Cytosines (DMCs) identified with various tools. Numbers within the heatmap (in the [0-100] range) represent the % of regions (or Cytosines) identified by the methods reported on the vertical axis overlapping (at least 1bp-overlap for the regions) with the regions (or Cytosines) identified by the methods reported on the horizontal axis. The numbers below the tool name indicate the number of DMRs or DMCs identified.

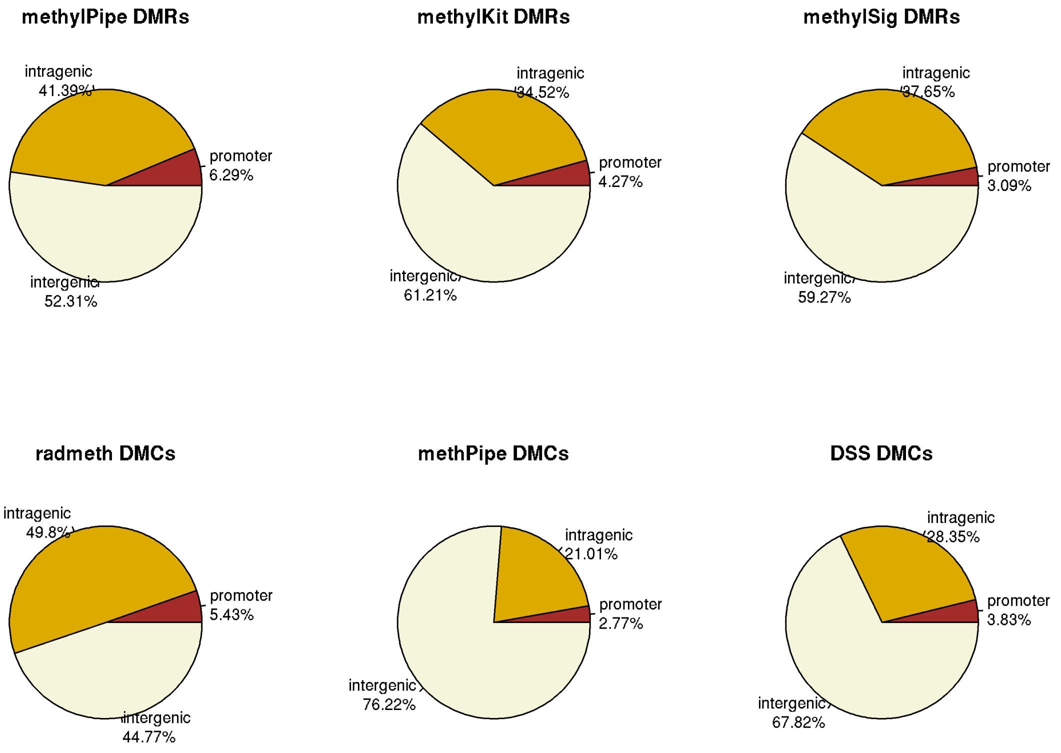

Figure 2 Annotation of differentially methylated regions (DMRs) and differentially methylated Cytosines (DMCs) identified with various tools. The percentage of regions and Cytosines assigned to promoter regions, intragenic and intergenic space are reported.

R source code used for the identification of the differentially methylated regions (DMRs) and differentially methylated Cytosines (DMCs) using various tools. The code used to make Figure 1 and 2 is also reported.

The input for all the programs is H1 and IMR90 cell line WGBS data modified according to the format required as input for each of the program. The data consists of "chromosome 1" only.

######## **methylPipe** #######

```
library(methylPipe)
library(BSgenome.Hsapiens.UCSC.hg18)
library(ListerEtAlBSseq)
h1data <- system.file('extdata', 'mc_h1_tabix.txt.gz', package='ListerEtAlBSseq')
h1uncov <- system.file('extdata', 'uncov_GR_h1.Rdata', package='ListerEtAlBSseq')
load(h1uncov)
H1.WGBS <- BSdata(file=h1data, uncov=uncov_GR_h1, org=Hsapiens)
imr90data <- system.file('extdata', 'mc_i90_tabix.txt.gz', package='ListerEtAlBSseq')
imr90uncov <- system.file('extdata', 'uncov_GR_imr90.Rdata', package='ListerEtAlBSseq')
load(imr90uncov)
IMR90.WGBS <- BSdata(file=imr90data, uncov=uncov_GR_imr90, org=Hsapiens)
H1.IMR90.set <- BSdataSet(org=Hsapiens, group=c("C","E"), IMR90_BS=IMR90.WGBS,
            H1_BS=H1.WGBS)
PMDs <- read.csv("IMR_PMDs.csv")
PMDs_gr <- GRanges(Rle(PMDs[,1]),IRanges(PMDs[,2],PMDs[,3]))
GRchr1 <- GRanges(Rle('chr1'), ranges=IRanges(start=1, end=length(Hsapiens$chr1)))
chr1_DMRs_12_1200 <- findDMR(object= H1.IMR90.set, ROI=GRchr1, Nproc=1,
             pmdGRanges=PMDs_gr, MCClass='mCG', coverage=5,
             dmrSize=12, dmrBp=1200, eprop=0.3)
hypo.DMRs.conso.chr1 <- consolidateDMRs(DmrGR=chr1_DMRs_12_1200, pvThr=0.05,
                 GAP=100, type="hypo", correct=TRUE)
hyper.DMRs.conso.chr1 <- consolidateDMRs(DmrGR=chr1_DMRs_12_1200, pvThr=0.05,
                 GAP=100, type="hyper", correct=TRUE)
methylPipe_DMRs <- append(hyper.DMRs.conso.chr1,hypo.DMRs.conso.chr1)
```

######## **methylKit** ##########

```
library(methylKit)
file.list <- list("mc_h1_1_MK.txt","mc_i90_1_MK.txt")
myobj <- read(file.list, sample.id=list("test","ctrl"), assembly="hg18", treatment=c(1,0),
context="CpG")
filtered.myobj <- filterByCoverage(myobj,lo.count=5,lo.perc=NULL,hi.count=NULL,hi.perc=99.9)
meth <- unite(filtered.myobj, destrand=FALSE)
head(meth)
tiles <- tileMethylCounts(meth,win.size=1000,step.size=1000)
myDiff <- calculateDiffMeth(tiles)
myDiff30p.hyper <- get.methylDiff(myDiff,difference=30,qvalue=0.05,type="hyper")
myDiff30p.hypo <- get.methylDiff(myDiff,difference=30,qvalue=0.05,type="hypo")
methylKit_DMRs <- get.methylDiff(myDiff,difference=30,qvalue=0.05)
```

######## **methylSig** ###########

```
### The same H1 and IMR90 data (chr1 only) is replicated three times ###
library(methylSig)
H1 <- "mc_h1_1_MK.txt"
I90 <- "mc_i90_1_MK.txt"
file.list <- c(H1,H1,H1,I90,I90,I90)
```

```
meth <- methylSigReadData(file.list, sample.id=c("test1","test2","test3","ctrl1","ctrl2","ctrl3"),
assembly="hg18", treatment=c(1,1,1,0,0,0), context="CpG")
methTile <- methylSigTile(meth,win.size = 100)
myDiffSigbothTile <- methylSigCalc(methTile, groups=c(1,0), min.per.group=3)
methylSig_res <- myDiffSigbothTile@results
ind <- which(methylSig_res[,2]< 0.05)
methylSig_gr <-
GRanges(myDiffSigbothTile@data.chr[ind],IRanges(myDiffSigbothTile@data.start[ind],myDiffSigb
othTile@data.end[ind]),meth=methylSig_res[ind,3])
methylSig_DMRs <- methylSig_gr[which(abs(methylSig_gr$meth) > 30)]
```

methPipe

The input data for this program (H1 and IMR90) is downloaded from the Methbase database
http://smithlabresearch.org/software/methbase/

```
~/methpipe/bin/methdiff -o H1_IMR.methdiff H1_methcount.meth IMR90_methcount.meth
methPipe <- fread("H1_IMR.methdiff")
ind <- which(methPipe$V5 < 0.05)
methPipe_sig <- methPipe[ind,]
mp_H1 <- (methPipe_sig$V6/(methPipe_sig$V6+methPipe_sig$V7))
mp_i90 <- (methPipe_sig$V8/(methPipe_sig$V8+methPipe_sig$V9))
mp_diff <- mp_H1-mp_i90
ind <- which(abs(mp_diff)>.3)
methPipe_sig <- methPipe[ind,]
methPipe_gr <-
GRanges(methPipe_sig$V1,IRanges(methPipe_sig$V2+1,methPipe_sig$V2+1),strand=methPipe_si
g$V3)
library(rtracklayer)
ch <- import.chain("/data/BA/kkishore/tools/hg19ToHg18.over.chain")
methPipe_DMCs <- liftOver(methPipe_new_gr,ch)
```

radMeth

control corresponds to H1 sample replicated three times (control_a, control_b, control_c) and case corresponds to IMR90 sample replicated three times (case_a, case_b, case_c)
design_matrix is made to define the six samples as control or case as per the specified input format of radMeth

```
~/radmeth/bin/make_table control_a control_b control_c case_a case_b case_c >
proportion_table.txt
~/radmeth/bin/wand -factor case design_matrix.txt proportion_table.txt > cpgs.bed
~/radmeth/bin/adjust -bins 1:100:1 cpgs.bed > cpgs.adjusted.bed
awk '$5 < 0.05 "{ print $0; $}"' cpgs.adjusted.bed > dm_cpgs.bed
radmeth <- fread("dm_cpgs.bed")
radmeth_DMCs <- GRanges(radmeth$V1,IRanges(radmeth$V2,radmeth$V3))
```

DSS

```
library(DSS)
require(bsseq)
library(data.table)
library(data.table)
h1 <- fread("mc_h1_DSS_header_chr1.txt")
i90 <- fread("mc_i90_DSS_header_chr1.txt")
h1 <- as.data.frame(h1)
```

```
BS1_DSS_chr1 <- makeBSseqData(list(h1,h1),paste("cond1",1:2,sep="."))
i90 <- as.data.frame(i90)
BS2_DSS_chr1 <- makeBSseqData(list(i90,i90),paste("cond2",1:2,sep="."))
dmls <- callDML(BS1_DSS_chr1,BS2_DSS_chr1)
save(dmls,file="dmls.Rdata")
ind <- which(dmls$fdr < 0.1)
dmls <- dmls[ind,]
DSS_DMCs <- GRanges(dmls$chr[ind],IRanges(dmls$pos[ind],dmls$pos[ind]))
```

combining all regions and computing **overlap** and **annotation**

```
library(compEpiTools)
require(TxDb.Hsapiens.UCSC.hg18.knownGene)
txdb <- TxDb.Hsapiens.UCSC.hg18.knownGene

All_DMRs_DMCs=list(methylPipe_DMRs=methylPipe_DMRs, methylKit_DMRs=methylKit_DMRs,
        methylSig_DMRs=methylSig_DMRs,radmeth_DMCs=radmeth_DMCs,
        methPipe_DMCs=methPipe_DMCs,DSS_DMCs=DSS_DMCs)
res <- overlapOfGRanges(GRlist=All_DMRs_DMCs, plot=TRUE)
methylPipe_anno <- GRannotateSimple(All_DMRs_DMCs$methylPipe_DMRs,txdb)
methylKit_anno <- GRannotateSimple(All_DMRs_DMCs$methylKit_DMRs,txdb)
methylSig_anno <- GRannotateSimple(All_DMRs_DMCs$methylSig_DMRs,txdb)
radmeth_anno <- GRannotateSimple(All_DMRs_DMCs$radmeth_DMCs,txdb)
methPipe_anno <- GRannotateSimple(All_DMRs_DMCs$methPipe_DMCs,txdb)
DSS_anno <- GRannotateSimple(All_DMRs_DMCs$DSS_DMCs,txdb)
methylPipe_anno_type <- sapply(methylPipe_anno, length)
MK_anno_type <- sapply(methylKit_anno, length)
methylSig_anno_type <- sapply(methylSig_anno, length)
radmeth_anno_type <- sapply(radmeth_anno, length)
methPipe_anno_type <- sapply(methPipe_anno, length)
DSS_anno_type <- sapply(DSS_anno, length)
methylPipe_anno_type <- round((methylPipe_anno_type/sum(methylPipe_anno_type))*100,2)
MK_anno_type <- round((MK_anno_type/sum(MK_anno_type))*100,2)
methylSig_anno_type <- round((methylSig_anno_type/sum(methylSig_anno_type))*100,2)
radmeth_anno_type <- round((radmeth_anno_type/sum(radmeth_anno_type))*100,2)
methPipe_anno_type <- round((methPipe_anno_type/sum(methPipe_anno_type))*100,2)
DSS_anno_type <- round((DSS_anno_type/sum(DSS_anno_type))*100,2)
png("DMRs_DMCs_Annotation.png",units="in", width=8, height=6, res=400)
col=c("brown","#ddaa00","beige")
par(mfrow=c(2,3))
labels <- paste0(names(methylPipe_anno_type),"\n", methylPipe_anno_type, "%")
pie(methylPipe_anno_type, main="methylPipe DMRs", labels=labels, col=col)
labels <- paste0(names(MK_anno_type),"\n", MK_anno_type, "%")
pie(MK_anno_type, main="methylKit DMRs", labels=labels, col=col)
labels <- paste0(names(methylSig_anno_type),"\n",methylSig_anno_type, "%")
pie(methylSig_anno_type, main="methylSig DMRs", labels=labels, col=col)
labels <- paste0(names(radmeth_anno_type),"\n", radmeth_anno_type, "%")
pie(radmeth_anno_type, main="radmeth DMCs", labels=labels, col=col)
labels <- paste0(names(methPipe_anno_type),"\n",methPipe_anno_type, "%")
pie(methPipe_anno_type, main="methPipe DMCs", labels=labels, col=col)
labels <- paste0(names(DSS_anno_type),"\n",DSS_anno_type, "%")
pie(DSS_anno_type, main="DSS DMCs", labels=labels, col=col)
dev.off()
```
